# Manipulating the Unfolded State of a Folded Protein through Site-Specific Backbone Modification

**DOI:** 10.1021/acs.biochem.5c00687

**Published:** 2026-03-03

**Authors:** Gabrielle E. Page, Yuhan Lin, W. Seth Horne

**Affiliations:** Department of Chemistry, 6614 University of Pittsburgh, Pittsburgh, Pennsylvania 15260, United States

## Abstract

Protein unfolded states are heterogeneous but can manifest local and long-range order. Replacement of side chains through site-directed mutagenesis is a common method to manipulate the unfolded state and elucidate its role in the folding process. Modification of the protein backbone represents a less explored complementary approach with the potential to elicit dramatic changes in conformational preferences from minimal chemical alteration. Prior work has shown backbone modification can affect unfolded ensembles as well as intrinsically disordered sequences. Here, we show that it can be used to rationally tune structural characteristics of the unfolded state of a folded protein. Using the GCN4 leucine zipper as a host, canonical α-residues throughout the chain are individually replaced by β^3^ or C_α_-Me-α analogues. The former modification enhances conformational freedom, the latter restricts it, and both retain the side chain at the substitution site. Characterization by circular dichroism and X-ray crystallography shows that the variants adopt folded structures identical to the prototype. Thermal and thermodynamic stability vary in complex ways with the context and nature of backbone modification; however, a uniform relationship is observed between substitution type and the sensitivity of folding free energy to chemical denaturant. This finding suggests systematic changes in solvent-accessible surface area of the unfolded ensemble among isomeric proteins differing only in the position of a single CH_2_ group. Collectively, these results demonstrate a platform for predictably tuning the properties of the unfolded state through minimal chemical modification, enabling new avenues for fundamental research on folding behavior of proteins as well as protein mimetics.

## Graphical abstract

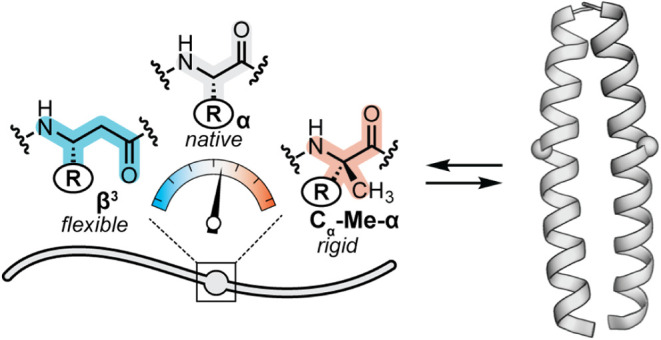

## Introduction

The immense range of three-dimensional folded conformations that can be specified by different sequences of amino acids in a polypeptide chain provides the foundation for protein structural and functional diversity in nature. The energetics of protein folding are complex and dictated by the combined effects of many weak noncovalent interactions involving the protein backbone, protein side chains, solvent water, and other molecules in the biological milieu. Beyond the folded conformation, which dominates for most sequences under physiological conditions, understanding the energetics of protein folding requires insight into the other side of the folding equilibrium—the unfolded state.^1^ Sparsely populated under native conditions for well-folded proteins, the unfolded state consists of an ensemble with a high degree of conformational heterogeneity; however, this ensemble is not necessarily random.^2^ Although challenging to study in molecular detail, methods including NMR,^3–6^ small-angle X-ray scattering,^7^ single-molecule FRET,^8–10^ and molecular dynamics (MD) simulation^11–15^ have yielded valuable information about the nature of nonnative protein states. Collectively, results of these efforts show secondary structure as well as tertiary contacts persist in the unfolded state of some systems. Further, reflecting a fundamental aspect of all protein chemistry, characteristics of the unfolded state are intimately tied to sequence as well as environment.^16^

One useful tool in the study of protein unfolded state ensembles is to manipulate their conformational preferences, which can reveal the role of local chain dynamics in folding energetics and folding pathways. A common strategy to this end is site-directed mutagenesis, where glycine substitution enhances local backbone flexibility, while introduction of alanine, proline, or a β-branched side chain restricts conformation toward a particular region of the Ramachandran plot.^17^ A complementary approach to side chain modification for engineering protein properties is to alter the chemical structure of the backbone. Beyond widespread application in construction of peptide and protein mimetics,^18,19^ backbone modification has also been explored to address fundamental questions about protein folding.^20–27^ In studies on the folding thermodynamics of artificial protein-like molecules, we have observed backbone modification can have significant effects on unfolded state and transition state ensembles.^28–30^ While this work was largely exploratory (i.e., making arbitrary modifications, then assessing their effects), Torbeev and coworkers have applied backbone modification in a rational manner to rigidify an intrinsically disordered protein and probe the role of localized conformational order in its biological function.^31^

Based on the above precedents, we envisioned the application of targeted backbone modification in the context of a well-folded protein to rationally tune the properties of the unfolded state ensemble. Our overall goal was a systematic and generalizable approach to either enhance or decrease protein backbone conformational freedom in a site-specific manner without changing side-chain composition or the structural characteristics of the folded state. Successful realization of such a technology would provide a useful tool to apply in the study of disordered protein states complementary to existing experimental and computational methods. We hypothesized the above goal could be achieved through the application of two closely related side-chain retaining substitutions for native α-residues: β^3^-residues and C_α_-Me-α-residues (Figure 1). These two monomers each involve the formal insertion of a CH_2_ group into a canonical α-residue and are thus regioisomeric to one another for a given side-chain identity. For the C_α_-Me-α-residue, the CH_2_ group is inserted between C_α_ and H_α_; the resulting geminal substitution at C_α_ restricts accessible backbone dihedral angles. For the β^3‑^residue, the CH_2_ group is inserted between C_α_ and C=O; this adds a new freely rotatable bond in the backbone, enhancing conformational flexibility. In work on the development of heterogeneous-backbone mimics of folded proteins, both β^3^ and C_α_-Me-α-residues have been shown to be well accommodated in diverse helical secondary structure contexts.^18,19^ Given the distinct conformational properties of these isomeric monomers, we set out to test the central hypothesis that they could be used in concert to site-specifically tune the characteristics of the unfolded state ensemble of a helical protein without measurable effects on its folded structure.

**Figure 1:**
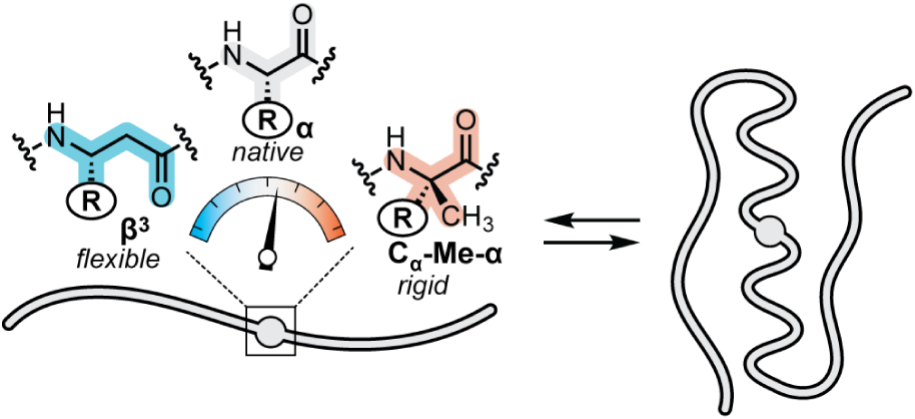
Schematic illustrating the central hypothesis underlying the present study. Site-specific backbone modification by replacement of an α-residue with a β^3^ or C_α_-Me-α analogue in a helical region of a folded protein tunes conformational freedom of the unfolded state ensemble with minimal impact on the folded structure.

## Materials and Methods

### Peptide Synthesis and Purification

GCN4-p1 prototype sequence **p1** (UniProt ID: P03069; M2V/Y17W) and its backbone-modified variants were synthesized by automated Fmoc solid-phase methods on a PurePep Chorus instrument (Gyros Protein Technologies) using TentaGel XV Rink Amide resin (0.24 mmol/g loading, Rapp Polymere) at a 0.025 mmol scale. Prior to the start of the synthesis, resin was allowed to swell in DCM for 30 min, then in DMF for 10 min. Fmoc deprotection was carried out by treatment of resin with 20% v/v 4-methylpiperidine/DMF for 5 min at room temperature and repeated with fresh reagents. Coupling reactions were performed by sequential delivery of the following reagents from the indicated stock solutions to the reaction vessel: Fmoc-protected amino acid (7.5 equiv, 0.25 M in DMF with 0.25 M OxymaPure), PyOxim (8.25 equiv, 0.275 M in DMF), and DIEA (15 equiv, 1.5 M in DMF). The final coupling reaction mixture was 0.11 M concentration protected monomer. The coupling reaction was allowed to proceed for 30 min at room temperature and then repeated with fresh reagents (i.e., double coupling at every cycle). Resin was washed with DMF (3×) after each coupling or deprotection. Following assembly of the desired full-length peptide chain, the N-terminus was acetyl capped by treatment with a freshly prepared solution of 8:2:1 v/v/v DMF/DIEA/Ac_2_O for 5–10 min at room temperature. Resin was transferred to a fritted syringe, washed sequentially with DMF (3×), DCM (3×), and MeOH (3×), then dried under vacuum. Cleavage of peptide from resin was accomplished by treatment with a solution of 95:2.5:2.5 v/v/v TFA/H_2_O/triisopropylsilane for 4 h at room temperature. The mixture was filtered, the resin washed with additional TFA, and the combined filtrates reduced to about half the initial volume under a stream of nitrogen. Peptide was precipitated by addition of 40 mL of cold diethyl ether. The mixture was centrifuged, the liquid decanted, and the pellet washed with an additional 40 mL of cold ether. After a second centrifugation and decanting, the pellet was dried under vacuum. Each crude peptide was dissolved in a mixture of solvent A (0.1% TFA in water)/solvent B (0.1% TFA in acetonitrile) and sonicated for 30–60 min to effect complete decarboxylation of the indole carbamic acid formed at the Trp­(Boc) residue during cleavage. Peptides were purified by preparative HPLC on a Waters LCMS system equipped with a Phenomenex Jupiter C18 column (250 × 21.2 mm, 300 Å pore size, 10 μm particle size) using gradients between solvents A and B. Nominal mass was confirmed by an ESI detector on the prep LCMS. Pooled fractions containing the purified product were lyophilized, and then further assessed by analytical HPLC (Phenomenex Jupiter C18 column, 250 × 4.6 mm, 300 Å pore size, 5 μm particle size) and HRMS (Thermo Fisher Q Exactive Orbitrap instrument) to confirm purity and identity (Figures S1–S15).

### Circular Dichroism Scans and Thermal Melts

Circular dichroism experiments were conducted on an Olis DSM 17 spectrophotometer equipped with a six-cell sample changer and Peltier temperature controller using 1 mm path length quartz cuvettes. Peptide stock solutions were prepared in water and concentration determined by UV absorbance (ε_280_ = 5690 M^–1^ cm^–1^ for the single Trp in each sequence).^32^ For scans and thermal melts, samples consisted of 50 μM peptide in 20 mM phosphate buffer pH 7. Scans were recorded at 20 °C from 195 to 260 nm in 1 nm increments with a 2 nm bandwidth and 3 s averaging time. For thermal melts under benign buffer conditions, ellipticity was monitored at 220 nm at temperatures ranging from 4 to 98 °C in 2 °C increments with a 0.05 °C deadband and 2 min equilibration time at each temperature. These data were fit to a two-state folding model using GraphPad Prism to obtain the midpoint of the thermal unfolding transition.^33^ In the fit, the slope of the unfolded baseline as a function of temperature and heat capacity change of unfolding (ΔC_p_) were set to zero.

### Analysis of Folding Thermodynamics by Coupled Thermal and Chemical Denaturation

A set of 12 samples were prepared for each variant composed of 50 μM peptide in 20 mM phosphate buffer pH 7 with varying urea concentration from 0 to 8 M. Concentration of urea stock solutions was quantified by refractive index.^34^ The exact urea concentration range employed for each sequence was determined based on stability observed in pilot measurements to maximize sampling of the unfolding curve. For each sample, molar ellipticity was recorded at 220 or 222 nm at a set of 6–7 temperatures ranging from 4 to 5 °C up to approximately the *T*_m_ observed for that variant in the absence of urea. A measurement at a reference temperature of 25 °C was included for all peptides. Equilibration time was 5 min at each temperature with a deadband of 0.05 °C.

Data were fit to a two-state monomer–dimer folding model, following derivations described.^35–37^ Briefly, each peptide was assumed to exist in equilibrium between a folded coiled coil dimer (*N*_2_) and an unfolded monomer (*U*):

1 $$N_{2}⇄2U$$

The equilibrium constant (*K*) for this equilibrium is given by

2 $$K=e^{\left(\frac{\Delta G^{H_{2}O}-m\left[urea\right]}{RT}\right)}$$

where $\Delta G^{H_{2}O}$ is the unfolding free energy in the absence of urea and *m* is the linear dependence of the observed free energy on denaturant concentration. The fraction unfolded peptide (*F*_*U*_) is given by

3 $$F_{U=}\frac{\sqrt{K^{2}+8P_{tot}K}-K}{4P_{tot}}$$

where *P*_*tot*_ is the total peptide concentration expressed in terms of monomer. Finally, the observed CD signal ([Θ]_*obs*_) at a given temperature and urea concentration follows:

4 $${[\Theta ]}_{obs}=F_{U}\left({[\Theta ]}_{U}+a\left[urea\right]+bT\right)+\left(1-F_{U}\right)\left({[\Theta ]}_{F}+c\left[urea\right]+dT\right)$$

where [Θ]_*U*_ and [Θ]_*F*_ are the molar ellipticities of the unfolded and folded states and parameters *a*, *b*, *c*, and *d* are the linear dependence of unfolded or folded baseline as a function of temperature or denaturant concentration.

For each peptide, CD data obtained as a function of temperature and urea concentration were globally fit to eqs 2–4 using GraphPad Prism with the following as floating parameters: $\Delta G^{H_{2}O}$ (one value for each temperature), *m* (shared across the data set), and baseline parameters [Θ]*_U_*, [Θ]_*F*_, *b*, *c*, and *d* (shared across the data set; *a* was constrained to zero). Values obtained for $\Delta G^{H_{2}O}$ at 298 K (Δ*G*°) and *m* are reported in Table 1, and individual fits for all peptides are shown in the Supporting Information (Figures S18–S19).

**Table 1:** Thermodynamic Parameters for Unfolding of **p1** and Backbone-Modified Variants^a^

	*T*_m_ (°C)	ΔG° (kcal mol^–1^)	*m* (kcal mol^–1^M^–1^)	ΔH**°** (kcal mol^–1^)	ΔS**°** (cal mol^–1^ K^–1^)	ΔC_p_ (kcal mol^–1^ K^–1^)
**p1**	59.9 ± 0.3	10.28 ± 0.09	0.84 ± 0.02	25.6 ± 0.2	51.5 ± 0.6	1.06 ± 0.02
**β^3^D7**	48.3 ± 0.2	8.56 ± 0.09	0.94 ± 0.03	25.8 ± 0.1	57.7 ± 0.5	1.08 ± 0.02
**α^Me^D7**	57.1 ± 0.3	8.98 ± 0.1	0.65 ± 0.02	19.9 ± 0.2	36.5 ± 0.8	0.77 ± 0.03
**β^3^E11**	34.4 ± 0.4	-	-	-	-	-
**α^Me^E11**	71.1 ± 0.2	11.78 ± 0.2	0.62 ± 0.03	26.1 ± 0.3	48.0 ± 1	0.92 ± 0.02
**β^3^S14**	57.3 ± 0.2	10.00 ± 0.09	0.88 ± 0.02	26.0 ± 0.2	53.7 ± 0.6	1.09 ± 0.02
**α^Me^S14**	63.6 ± 0.3	11.13 ± 0.12	0.76 ± 0.02	26.0 ± 0.2	50.0 ± 0.8	1.17 ± 0.03
**β^3^E20**	38.9 ± 0.9	7.32 ± 0.05	0.90 ± 0.02	26.6 ± 0.3	64.6 ± 0.8	0.89 ± 0.04
**α^Me^E20**	53.0 ± 0.4	9.48 ± 0.08	0.72 ± 0.02	23.8 ± 0.2	48.0 ± 0.8	0.98 ± 0.03
**β^3^A24**	39.5 ± 0.5	7.67 ± 0.06	0.97 ± 0.03	27.4 ± 0.3	66.2 ± 1.2	0.85 ± 0.06
**α^Me^A24**	54.3 ± 0.3	9.26 ± 0.09	0.68 ± 0.02	23.7 ± 0.3	48.5 ± 0.9	0.92 ± 0.03
**β^3^K28**	46.3 ± 0.3	8.52 ± 0.08	1.00 ± 0.03	26.7 ± 0.2	61.0 ± 0.6	1.06 ± 0.03
**α^Me^K28**	58.5 ± 0.2	9.92 ± 0.13	0.66 ± 0.02	24.9 ± 0.3	50.1 ± 0.9	0.87 ± 0.03
**α^Me^D7/α^Me^K28**	62.9 ± 0.4	8.97 ± 0.13	0.53 ± 0.03	19.5 ± 0.8	35.6 ± 2.8	0.56 ± 0.06
**α^Me^E11/α^Me^S14**	77.4 ± 0.2	12.43 ± 0.29	0.57 ± 0.03	21.4 ± 1.2	30.1 ± 3.9	0.98 ± 0.06

a Data obtained from CD experiments monitored as a function of temperature and added urea as a denaturant. Reported uncertainties are standard error from the fits. See methods for details.

Variation of observed Δ*G* as a function of temperature from the above analysis was fit to the Gibbs–Helmholtz equation:^38^

5 $$\Delta G=\Delta H^{{}^{\circ}}-T\Delta S^{{}^{\circ}}+\Delta C_{p}\left(T-T^{{}^{\circ}}-T\ln\left(\frac{T}{T^{{}^{\circ}}}\right)\right)$$

to obtain change in enthalpy, entropy, and heat capacity associated with unfolding (Δ*H*°, ΔS°, and Δ*C_p_*, respectively). These values are also reported at a reference temperature (*T*°) of 298 K (Table 1).

### Crystallization, Diffraction Data Collection, and Structure Determination

For a subset of the peptides (**p1**, **α^Me^E11**, **β^3^E20**, **β^3^A24**, **α^Me^A24**, **β^3^K28**, **α^Me^K28**), high-resolution structures were determined by X-ray crystallography. Single crystals of each peptide were grown by hanging drop vapor diffusion from stock solutions of ∼2 mM peptide in water. In a typical experiment, drops were prepared by mixing 0.7 μL of peptide stock with 0.7 μL of crystallization buffer and allowed to equilibrate at room temperature over a well containing 0.7 mL of buffer. Buffer composition yielding the crystals used for diffraction analysis varied with sequence but centered around a small range of conditions: 0.2–0.3 M sodium acetate, 0.1 M sodium citrate, pH 4.6–5.6, 5–15% w/v PEG 3350. Exact buffers used to crystallize each variant are provided in the Supporting Information (Table S1). Diffraction data were collected using Cu K_α_ radiation on a Bruker D8 VENTURE diffractometer equipped with an IμS 3.0 microfocus sealed-tube X-ray source, HELIOS multilayer Montel optics, PHOTON III detector, and Oxford Cryostream 1000 operated at 100 or 150 K. A single crystal of each peptide was cryo protected by a brief soak in crystallization buffer containing 10–15% glycerol (Table S1), harvested in a nylon loop, and frozen by rapid transfer to the cold stream. Raw diffraction images were indexed, integrated, and scaled using XDS (Tables S2–S3).^39^ The structure for **p1** was solved by molecular replacement with a published X-ray structure of GCN4-p1 (PDB 4DMD) as the search model.^40^ Structures for variants were solved by molecular replacement with the refined structure of p1 above as the search model. Molecular replacement and automated refinement were carried out using the Phenix software suite,^41^ and Coot was used for manual model building. Coordinates and structure factors are deposited in the PDB under accession codes 9Z1P (**p1**), 9Z1Q (**α^Me^E11**), 9Z1R (**β^3^E20**), 9Z1S (**β^3^A24**), 9Z1T (**α^Me^A24**), 9Z1U (**β^3^K28**), and 9Z1V (**α^Me^K28**).

## Results and Discussion

### Design and Synthesis of Single-Site Substitution Variants

As a system to test the envisioned strategy to manipulate the unfolded state of a well-folded protein through backbone modification, we selected the dimeric α-helical coiled coil GCN4-p1.^42–44^ The exact prototype sequence employed is a double mutant of GCN4-p1 with two substitutions relative to the natural peptide: M2V and Y17W (**p1**, Figure 2). The former modification eliminates potential complications from methionine oxidation during storage and analysis,^37^ while the latter provides a sensitive chromophore that also enables fluorescence-based measurements. Coiled-coil forming peptides like GCN4-p1 are characterized by a seven-residue “heptad” repeat, with sequence positions denoted *abcdefg*. Heptad positions *a* and *d* in coiled coils bear predominantly hydrophobic side chains and form the buried core in the assembled quaternary structure. Heptad positions *e* and *g* are peripheral to the coiled coil interface and sometimes engage in interchain salt bridges that help stabilize the fold. Finally, heptad positions *b*, *c*, and *f* are solvent exposed and project away from the interchain interface in the folded state. We designed a series of single-site backbone modification variants of **p1** in which an α→β^3^ or α→C_α_-Me-α substitution was made at one of six solvent-exposed sites in the sequence. In each variant, the side chain of the replaced α-residue is retained in the artificial C_α_-Me-α or β^3^ monomer that replaces it. Three modification sites are in the N-terminal half of the chain (D7, E11, S14) and three in the C-terminal half (E20, A24, K28). In terms of the heptad repeat, five sites occupy a solvent-exposed *b*, *c*, or *f* position and the sixth an interfacial *e* position. The above design considerations yielded a set of 12 variants of **p1** (6 modification sites × 2 substitutions each). These variants are all isomeric with respect to each other and differ only in the placement of a single CH_2_ group in the 4 kDa macromolecule. Variant nomenclature combines the modification type and site (i.e., **β^3^D7** indicates the peptide with a side-chain-retaining α→β^3^ substitution at Asp7).

**Figure 2:**
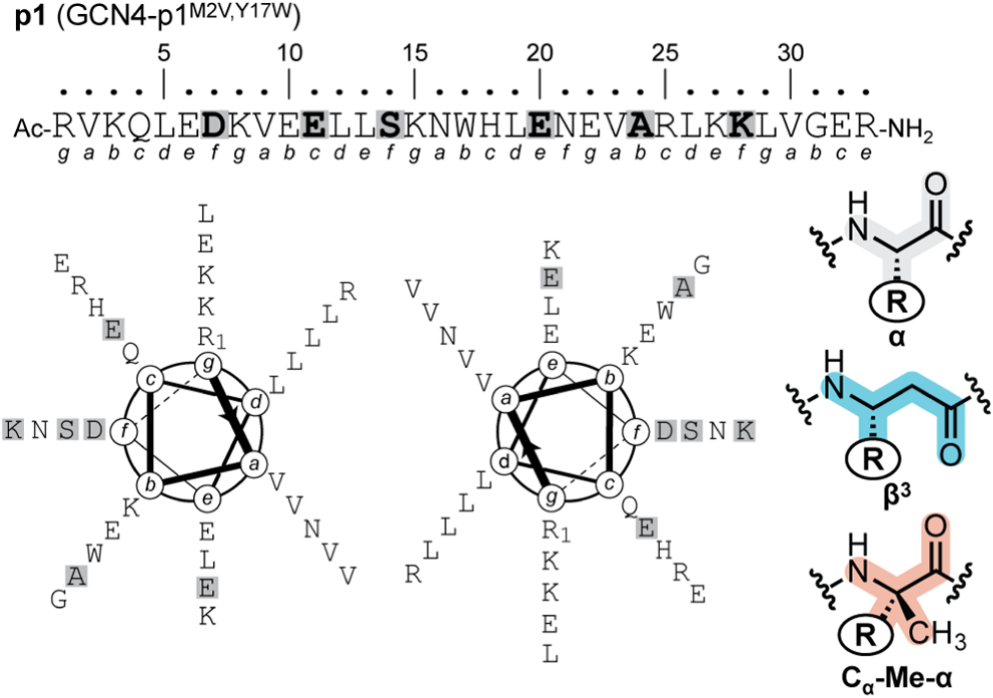
Sequence of GCN4-p1^M2V,Y17W^ (**p1**) with heptad positions labeled and helical wheel diagram mapping the **p1** sequence in a coiled coil dimer. Residues highlighted in gray indicate sites targeted for backbone modification. At each of these positions, the α-residue in the prototype was substituted by either a β^3^ or C_α_-Me-α analogue with the same side chain as the replaced α-residue.

Prototype peptide **p1** and backbone-modified variants were synthesized by Fmoc solid-phase methods, purified by preparative HPLC, and the identity and purity of isolated material confirmed by analytical HPLC and ESI-MS. Synthesis was uneventful for the most part and crude purities almost uniformly high. A notable exception was the variant containing a C_α_-Me-α-Ser residue (**α^Me^S14**). For this peptide, the crude material obtained after cleavage from resin showed two significant components in the HPLC chromatogram, both with mass corresponding to the expected product (Figure S16). These species were readily separable; however, the situation posed the vexing question of which corresponded to the desired product and what process had produced the byproduct. Based on our previous experience with synthetic protein mimetics containing the sterically hindered monomer N-Me-Thr,^45^ we hypothesized the second peak may arise from N→O acyl transfer at C_α_-Me-α-Ser under the strongly acidic conditions of neat TFA.^46^ To test this hypothesis, we subjected the crude mixture to pH ∼ 8.5 and observed complete conversion of the minor isomer to the major (Figure S16). This finding is consistent with the known rapid rearrangement of isoacyl depsipeptides to peptides under mild basic conditions^47–49^ and supports our hypothesis as to the origin of the byproduct and identity of the isolated product.

### Impacts of Backbone Modification on Folded State Structure

To assess impacts of backbone alteration on coiled coil folded structure, we first analyzed the prototype and single-site substitution variants by circular dichroism (CD) spectroscopy. A CD scan acquired at 20 °C for **p1** at 50 μM concentration in 20 mM phosphate buffer at pH 7 shows minima at 208 and 220 nm, consistent with almost entirely α-helical secondary structure content (Figure 3). CD spectra for the C_α_-Me-α variants match the prototype within uncertainty, supporting the innocuous nature of this modification with respect to folded structure. CD scans for the β^3^ variants show similar double minima but with a subtle shift in the ratio of the peaks favoring the signal at 208 nm. This is a well-documented phenomenon for heterogeneous backbones containing mixtures of α and β^3^ residues in a well-folded helical conformation.^28,45,50^ Thus, the CD results for the series are consistent with similar folded states among the prototype and most variants. The exception is **β^3^E11**, where the reduced magnitude of both CD peaks suggests the variant is not fully folded at room temperature.

**Figure 3:**
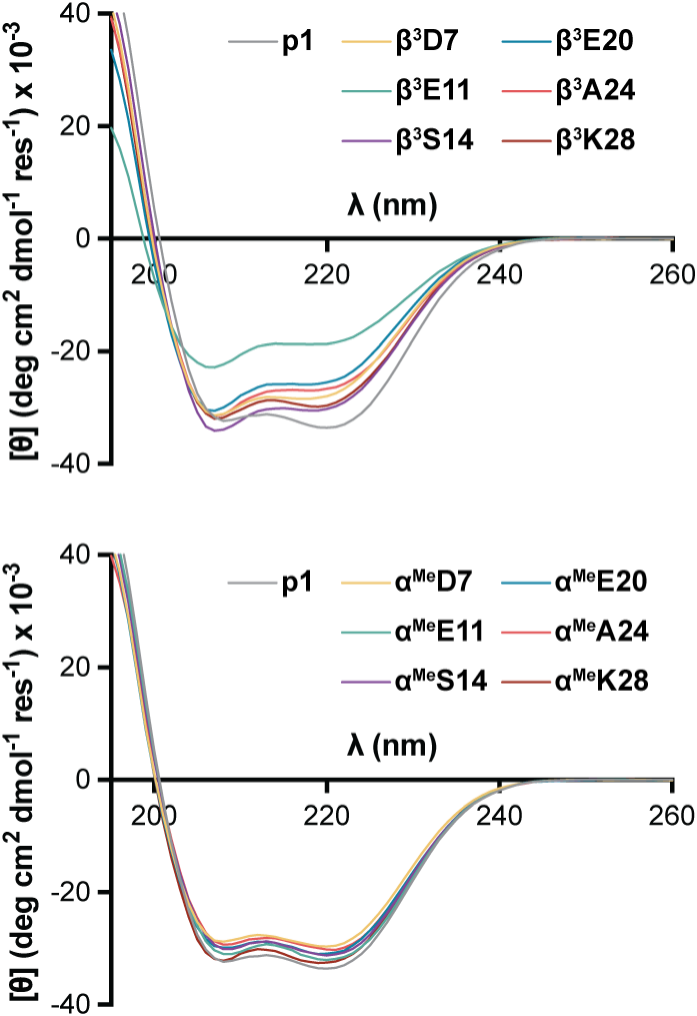
Circular dichroism scans for **p1** and 12 single-site backbone modified variants. Conditions: 50 μM peptide in 20 mM phosphate, pH 7 at 20 °C.

To corroborate the findings from the CD scans as well as evaluate the degree of folded structure similarity between the prototype and backbone-modified variants at a higher level of precision, we pursued X-ray crystallographic analysis. Prototype **p1** and the full set of single-site variants were subjected to crystallization screens by hanging drop vapor diffusion under a narrow range of conditions around a previously reported buffer used for the wild-type GCN4-p1 dimer (acetate and citrate at pH ∼ 5 with polyethylene glycol 3350).^40^ This limited screen readily yielded diffraction quality crystals of prototype **p1**, three C_α_-Me-α residue containing analogues (**α^Me^E11**, **α^Me^A24**, **α^Me^K28**), and three β residue containing analogues (**β^3^E20**, **β^3^A24**, **β^3^K28**). X-ray diffraction data were collected on a single crystal of each of these peptides, and the corresponding structures solved by molecular replacement using a published structure of GCN4-p1. In most instances, the asymmetric unit was a coiled coil dimer; however, the lattice of **β^3^E20** contained two dimers related by noncrystallographic symmetry (0.12 Å backbone RMSD). Structures were refined to 1.5–1.9 Å resolution (Tables S2–S3).

The X-ray structure results show that the prototype and each of the six variants adopt dimeric coiled-coil folds almost identical to that of GCN4-p1 (Figure 4A). The backbone RMSD for overlay of **p1** with a previously reported crystal structure of GCN4-p1 is small (0.45 Å for residues 1–30), and the variants show a similar level of agreement with the **p1** prototype (0.23–0.49 Å). The resolution of the diffraction data reveals detailed structural features in the electron density maps (Figure 4B), providing insight into local conformational behavior of the artificial residues as well as rotameric preferences of their proteinogenic side chains. While the sites selected for backbone modification are peripheral or solvent-exposed in the coiled coil, some polar residues at these positions engage in salt bridges. Three of the four polar residues modified (Asp7, Glu11, and Glu20) are observed to form an intra- or interchain salt bridge in the crystal structure of prototype p1. To assess the potential effects of backbone modification on this network of ionic interactions, we compared the polar contacts of side chains from backbone modified residues in the crystal structures of **α^Me^E11** and **β^3^E20** to the corresponding α-residue in the prototype. These results show that both α→β^3^ and α→C_α_-Me-α substitution maintains the polar contact (Figure 4C), supporting the utility of side-chain retaining modifications to isolate changes in molecular properties to backbone conformational freedom.

**Figure 4:**
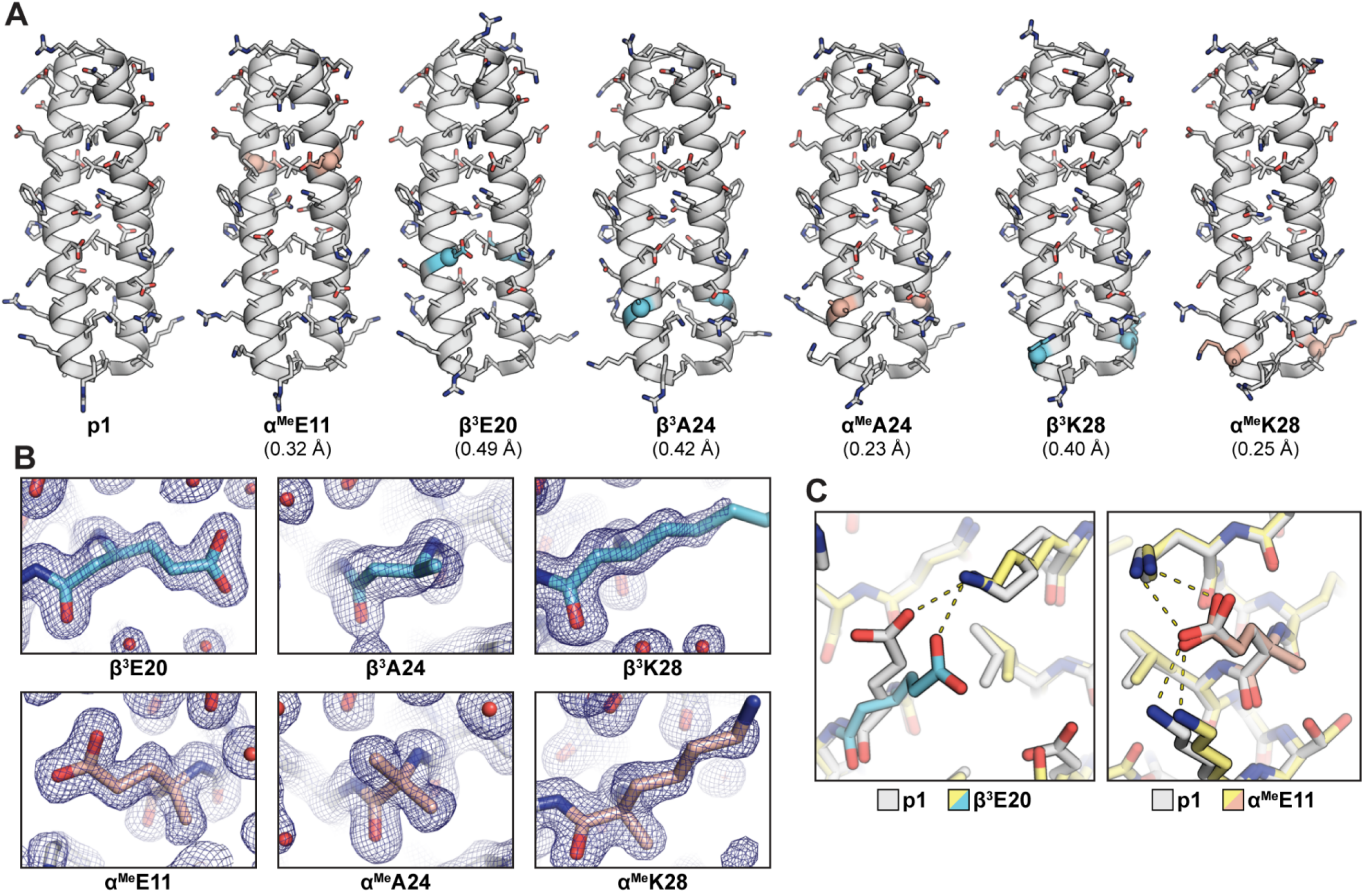
X-ray crystal structures of **p1** and six backbone-modified variants. (A) Cartoon representation of the coiled coil quaternary fold with side chains shown as sticks. Backbone modification sites are colored according to the scheme in Figure 2, and residues 31–33 from the disordered tail are omitted. Backbone RMSD for overlay with **p1** is shown in parentheses. (B) Views of 2F_o_–F_c_ electron density maps contoured at 1 σ around an artificial residue from each indicated variant. (C) Comparison of salt bridges involving polar side chains near the backbone modification sites in **β^3^E20** and **α^Me^E11** to corresponding interactions in **p1**.

### Impacts of Backbone Modification on Folding Thermodynamics and Unfolded State Properties

Having established site-specific backbone engineering in GCN4-p1 has minimal effect on its folded structure, we next sought to determine impacts of altered backbone conformational freedom on folding energetics. We first evaluated thermal conformational stability of **p1** and variants by variable temperature CD, monitoring the molar ellipticity at 220 nm from 4 to 98 °C for samples consisting of 50 μM peptide in 20 mM phosphate pH 7 (Figures 5A, S17). All variants show cooperative unfolding transitions except for β^3^E11, which lacks a well-defined folded baseline. Taken with the CD scan results above, this observation suggests that backbone modification is too destabilizing for **β^3^E11** to allow for a fully folded state within the temperature range of the experiment. The midpoint of the thermal unfolding transition (*T*_m_) for the prototype p1 was 60 °C, and *T*_m_ values for the variants ranged from 34 to 71 °C (Δ*T*_m_ −25 °C to +11 °C relative to **p1**; Figure 5B, Table 1). With respect to backbone modification type, every β^3^ residue containing variant was less stable than the corresponding isomeric variant with a C_α_-Me-α monomer at the same site. In terms of the context for backbone modification, thermal stability effects of a given monomer type varied with sequence. Most variants have a lower *T*_m_ than the prototype; however, two analogues (**α^Me^E11**, **α^Me^S14**) are more thermally stable, and one other (**α^Me^K28**) is similar within uncertainty. Taken with the finding of virtually identical folded structures in the X-ray crystallographic analysis, the thermal stability results for the isomeric coiled coil peptides show that small changes in backbone composition can have large impacts on the energetics of the folding equilibrium.

**Figure 5:**
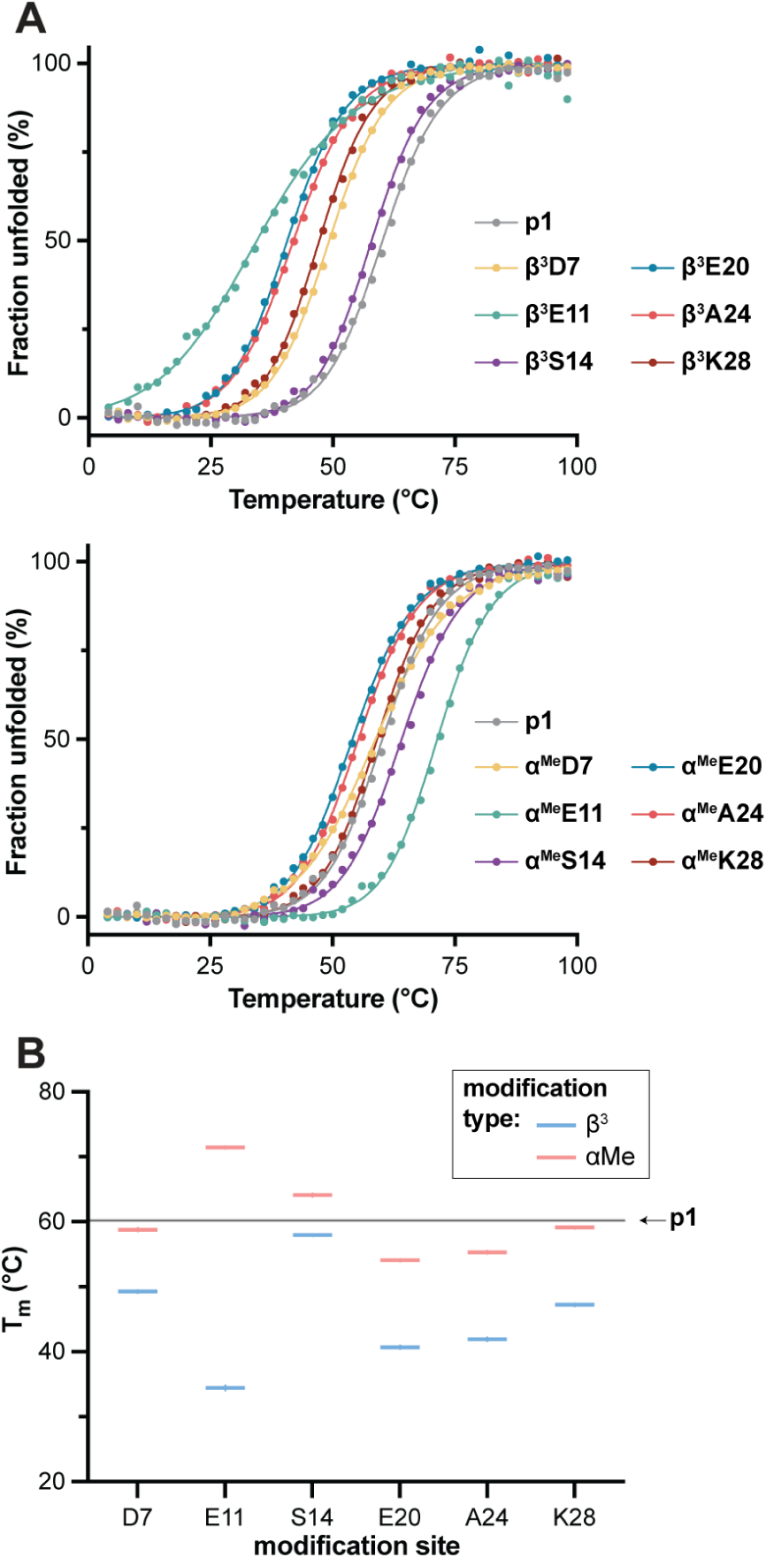
(A) Circular dichroism thermal melts for **p1** and 12 single-site backbone modified variants. Conditions: 50 μM peptide in 20 mM phosphate, pH 7 at 20 °C. Points depict observed molar ellipticity after population normalization and the lines show fits to a two-state folding model. Corresponding data prior to population normalization are provided in Supporting Information (Figure S17). (B) Summary of thermal unfolding midpoint (*T*_m_) values determined from the fits. Uncertainties (shading around the horizontal line for **p1**, error bars for the variants) are standard errors from the fits.

To better understand the energetic basis for observed thermal stability differences and gain data bearing on the hypothesis of altered unfolded state properties, we next quantified the thermodynamic parameters for the folding equilibria of **p1** and each variant. This was again achieved by CD spectroscopy, now monitoring unfolding as a function of both temperature and concentration of added urea as a chemical denaturant. The experiment was designed to efficiently sample the unfolding transition across a range of temperatures while also capturing the fully folded and unfolded baselines. Samples matched conditions described above in peptide and buffer. Temperature was varied in 5–6 increments from 4 to 5 °C up to the approximately the *T*_m_ of the peptide; one measurement was included at 25 °C for every sequence to enable comparison at a common reference temperature. Urea concentration was varied in 10–12 steps in a range (0–4 M, 0–6 M, or 0–8 M) determined based on the degree of stability of each sequence toward chemical unfolding. Variant **β^3^E11** was excluded from the experiment due to its very low thermal stability.

Coupled thermal chemical denaturation data for each peptide were globally fit to a model for a two-state equilibrium consisting of unfolded monomer and folded dimer. This two-state model has been applied previously in biophysical analysis of folding thermodynamics for GCN4-p1 and mutants by spectroscopic as well as calorimetric methods.^37,51–55^ We reasoned the small changes among the isomeric variants under study would not alter the two-state folding mechanism of GCN4-p1; however, it is important to note that the data set in the present study does not provide an incisive test of this hypothesis. The above analysis provided the free energy of unfolding at each experimental temperature (ΔG, including ΔG° at the reference temperature of 25 °C) and the linear dependence of the unfolding free energy on denaturant concentration (*m*), which was assumed to be constant with temperature. A subsequent fit of the resulting temperature dependent ΔG values to the Gibbs–Helmholtz equation yielded the individual enthalpic and entropic contributions to unfolding (ΔH°, ΔS°) as well as the heat capacity change associated with unfolding (ΔC_p_). Representative fits are shown in Figure 6 and the remainder in the Supporting Information (Figures S18–S19); a complete listing of the thermodynamic parameters is provided in Table 1.

**Figure 6:**
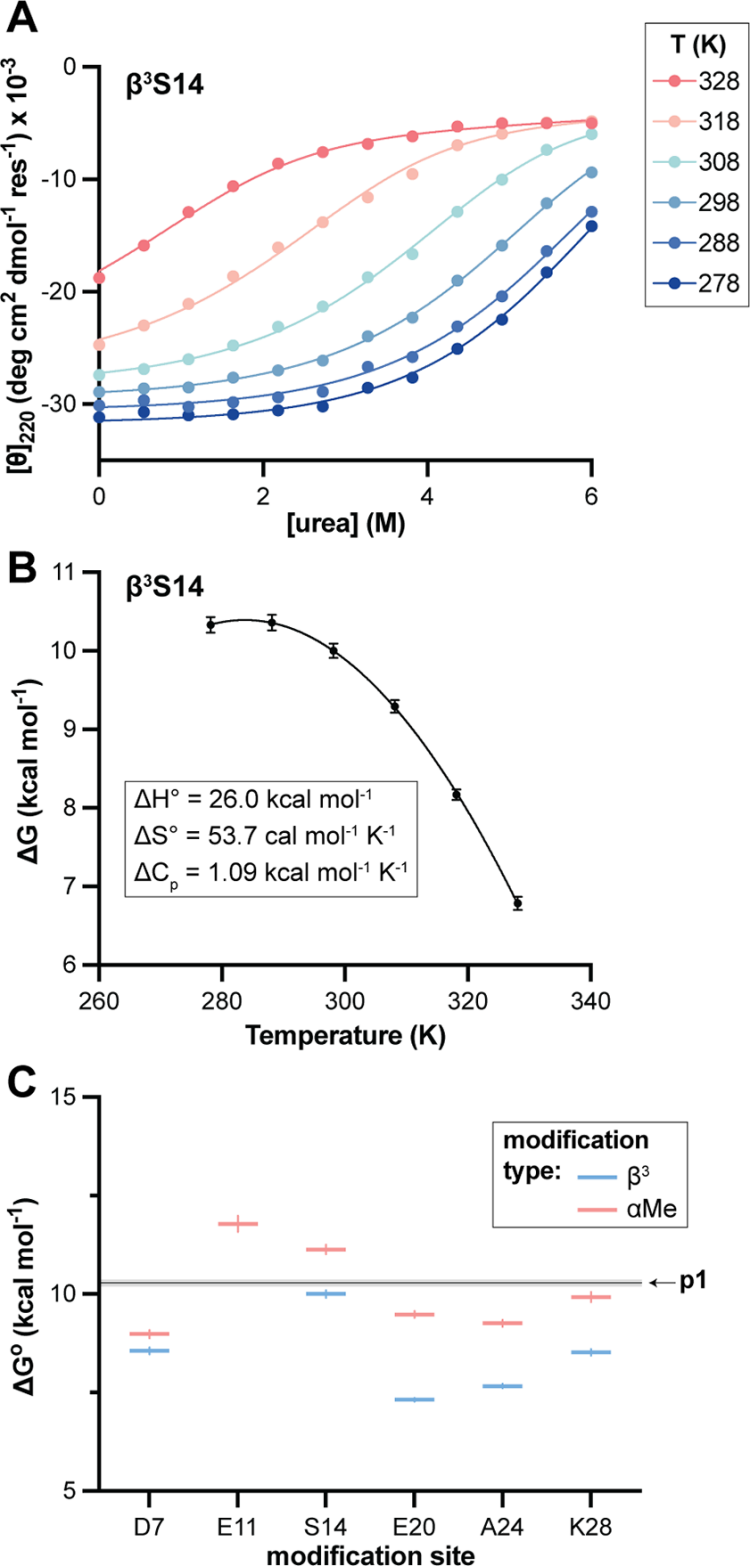
(A) Thermal/chemical denaturation monitored by circular dichroism for a representative **p1** variant (**β^3^S14**). Points depict experimentally observed molar ellipticity for a sample of 50 μM peptide in 20 mM phosphate pH 7 at the indicated temperature and urea concentration. Lines depict a global fit of the data set to a two-state monomer–dimer folding equilibrium. (B) Free energy of unfolding (ΔG) as a function of temperature for **β^3^S14**. Data points are determined from fits in (A) and error bars the parameter uncertainties from the fits. The line depicts a fit to the Gibbs–Helmholtz equation, which yields the enthalpy (ΔH°), entropy (ΔS°), and heat capacity change (ΔC_p_) associated with unfolding. (C) Summary of free energy of unfolding at 25 °C (ΔG°) for **p1** and 11 single-site variants. Uncertainties (shading around the horizontal line for **p1**, error bars for the variants) are standard errors from the fits. Data and fits for all peptides are provided in the Supporting Information.

The unfolding free energy for **p1** (GCN4-p1^M2V/Y17W^) determined from the analysis (10.3 kcal mol^–1^) is in good accord with prior reported values for similar sequences (9.9 kcal mol^–1^ for GCN4-p1^M2V^ and 10.5 kcal mol^–1^ for GCN4-p1^Y17W^).^37,54^ The effect of backbone modification on conformational stability varies significantly among the isomeric analogues (ΔΔG° from −3.0 to +1.5 kcal mol^–1^ relative to **p1**, where a more positive value corresponds to a more stable fold). Trends in ΔG° track with those noted above for *T*_m_; α→C_α_-Me-α substitution at a given position is consistently superior to α→β^3^ replacement at the same site, and both **α^Me^E11** and **α^Me^S14** are more thermodynamically stable than the prototype. Also apparent in the data is a pronounced importance of backbone modification context in determining its effect on ΔG° (Figure 6C). Inspecting the enthalpic and entropic origins of free energy differences reveals additional insights. All the β^3^ variants are entropically destabilized relative to the canonical backbone with a compensating enthalpic stabilization. The opposite holds for most C_α_-Me-α analogues, where backbone modification is entropically beneficial and enthalpically detrimental. The outliers are the two variants that are more thermodynamically stable than the prototype (**α^Me^E11** and **α^Me^S14**). In both these cases, backbone modification has a favorable effect on both enthalpy and entropy of folding. It is tempting to mine the above thermodynamic trends for insights related to the hypothesis that backbone engineering is impacting the unfolded state; however, enthalpy entropy compensation in protein folding is a complex phenomenon with many factors that confound its application to simply describe the properties unfolded state.^56^ Fortunately, another metric arising from the above experiments is more direct in its relationship to unfolded state properties.

The determination of conformational stability of proteins by the action of chemical denaturants, as employed in the present work, is a classical method in protein biophysics. The analysis of data from such experiments is predicated on the empirical observation that ΔG varies in a linear fashion with denaturant concentration (δΔG/δ­[denaturant] = *m*, as noted above). Early theoretical work postulated that the magnitude of *m* is proportional to the change in solvent-accessible surface area (SASA) accompanying the folding process (i.e., ΔSASA of the unfolded vs folded state).^57,58^ This was later validated experimentally through analyses of large collections of proteins with known structure and folding energetics.^36,59–61^ Given the single-site backbone-modified **p1** variants are isomeric and have virtually identical folded structures, it is reasonable to assume that changes in *m* across the series are likely to be dominated by altered properties of the unfolded state ensemble. Unlike the other thermodynamic parameters for the folding equilibria of **p1** and variants described above, effects of backbone modification on the magnitude of *m* have a clear and uniform relationship to substitution type (Figure 7). Relative to prototype **p1**, α→β^3^ replacement consistently increases *m*, while α→C_α_-Me-α substitution reduces it. To place the observed numerical differences in more concrete structural terms, an estimate based on one prior published analysis^60^ suggests the ΔSASA among the series varies by a factor of approximately 2-fold (from ∼2900 Å^2^ for **α^Me^E11** to ∼5800 Å^2^ for **β^3^K28**). It is important to note that these estimated ΔSASA values are predicated on the assumption that empirical relationships between *m* and ΔSASA observed for natural proteins hold for artificial backbones. Regardless of the exact effect on ΔSASA, changes in the *m* value observed with altered backbone composition provide strong support for these chemical changes influencing unfolded state properties.

**Figure 7:**
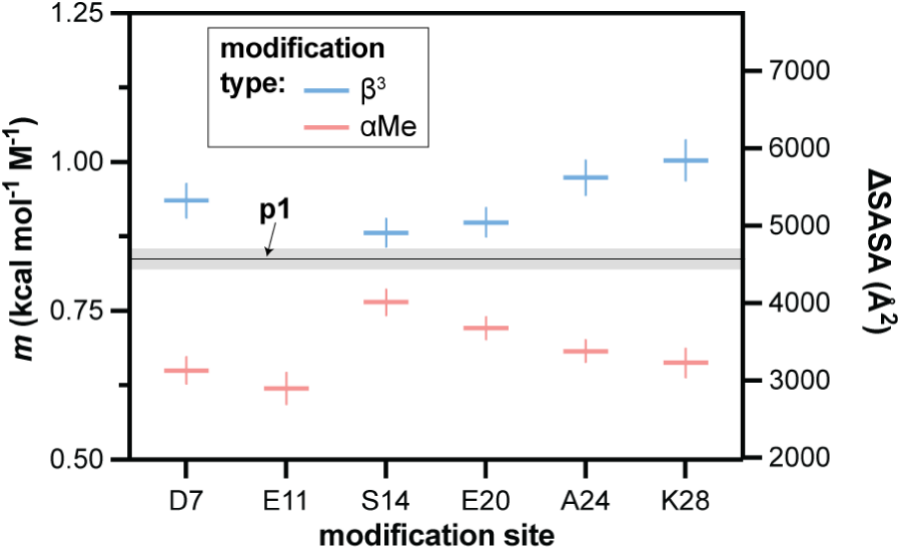
Summary of the linear variation of free energy of unfolding with urea concentration (*m*) for **p1** and 11 single-site variants. Uncertainties (shading around the horizontal line for **p1**, error bars for the variants) are standard errors from the fits. The right *y*-axis depicts an estimated solvent-accessible surface area change upon unfolding (ΔSASA) for the corresponding *m* value range; these estimated values were calculated according to the equation *m* = 243 + 0.13*ΔSASA (cal mol^–1^ M^–1^).^60^

### Design, Synthesis, and Characterization of Dual-Site Modified Analogues

Having obtained evidence supporting the ability to predictably control unfolded state solvation through single-site backbone modification with a β^3^ or C_α_-Me-α residue, we next examined the effects of mutually reinforcing modifications at multiple sites in the chain. As α→β^3^ residue substitution was consistently thermally destabilizing, we anticipated dual-site modification with this monomer would prove intractable in the biophysical experiments. Thus, we focused on dual α→C_α_-Me-α substitution. We designed two additional analogues of **p1** combining substitutions from single-site variants above. In one dual-site modified variant, modifications are incorporated near the termini (**α^Me^D7/α^Me^K28**), while in the other they are placed near the middle of the chain (**α^Me^E11/α^Me^S14**). These two peptides were synthesized and purified as described above, then subjected to analysis by CD. Scan results suggest the dual-site modified variants adopt similar folds as the prototype and other single-site variants (Figure 8A), and thermal melts show well-defined cooperative unfolding transitions (Figure 8B). Thermal conformational stability of **α^Me^E11/α^Me^S14** is substantially increased relative to prototype, while that of **α^Me^D7/α^Me^K28** is comparable to the canonical backbone (Δ*T*_m_ of +18 °C and +3 °C relative to **p1**, respectively). Coupled thermal chemical denaturation experiments in the presence of urea (Figure S19) show that the effect of backbone modification on ΔG° depends on context for the substitution. Trends in ΔG° for the dual-site variants track with behavior of the corresponding single-site modified analogues (Figure 8C). The folded conformational stability of **α^Me^D7/α^Me^K28** is lower than p1, as is the case for single-site variants **α^Me^D7** and **α^Me^K28**. In contrast, folded stability of **α^Me^E11/α^Me^S14** is higher than the prototype, as is the case for single-site variants **α^Me^E11** and **α^Me^S14**. The effect of backbone modification on *m* is consistent regardless of modification context, and the magnitude of the effect is greater for the dual-site variants, which show *m* values lower than that of **p1** or any single-site variant (Figure 8D). The changes correspond to estimated ΔSASA values reduced by a factor of 2-fold relative to the canonical backbone. This result shows that incorporating mutually reinforcing modifications at multiple sites in the chain exerts a greater effect on the unfolded state ensemble than single-site substitution.

**Figure 8:**
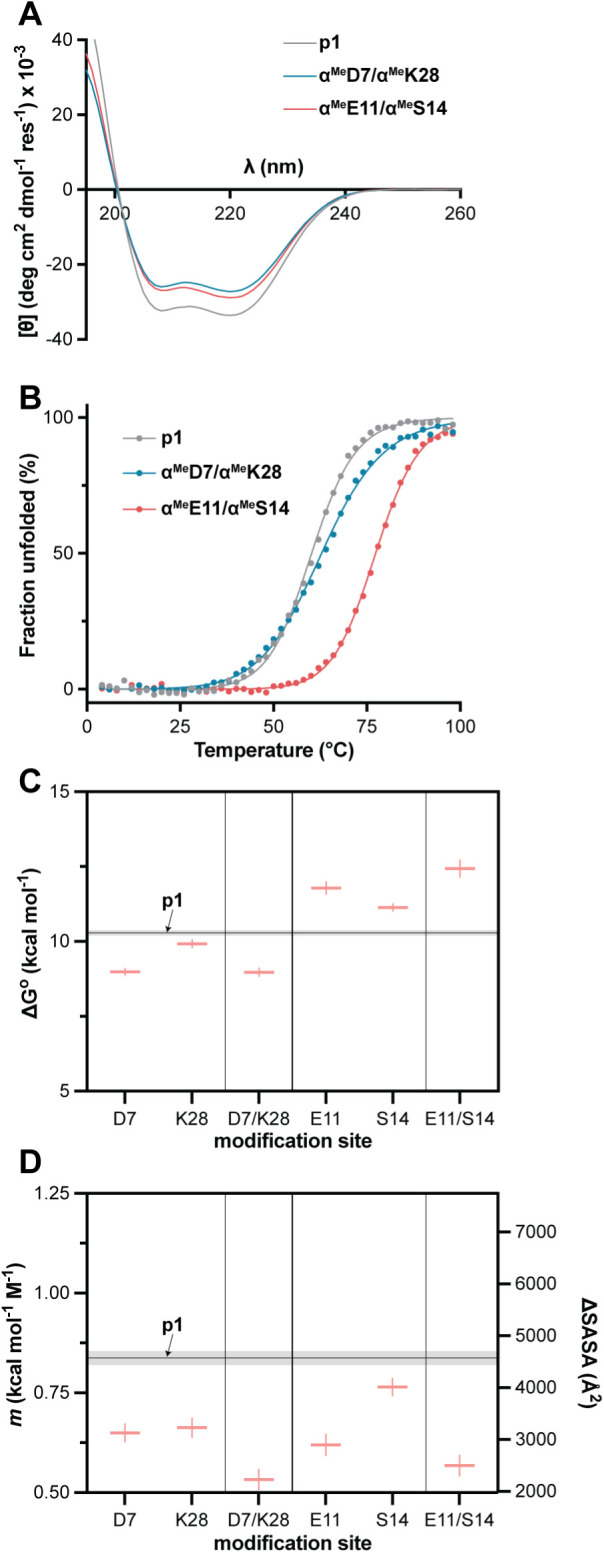
(A) CD scans at 20° for p1 and dual site modified variants α^Me^D7/α^Me^K28 and α^Me^E11/α^Me^S14. Conditions: 50 μM peptide in 20 mM phosphate, pH 7. (B) CD thermal melts under conditions as in (A). Points depict observed molar ellipticity after population normalization and lines show fits to a two-state folding model. Corresponding data prior to population normalization are provided in Supporting Information (Figure S17). (C, D) Summary of ΔG° and *m* values obtained by coupled thermal/chemical denaturation for the dual site modified variants compared to **p1** and corresponding single-site analogues. The right *y*-axis in the *m* value plot depicts an estimated solvent-accessible surface area change upon unfolding (ΔSASA) for the corresponding *m* value range; these estimated values were calculated according to the equation *m* = 243 + 0.13*ΔSASA (cal mol^–1^ M^–1^).^60^

## Conclusions

In summary, we have described here a method that can be applied to manipulate the conformational properties of the unfolded state ensemble of a folded protein through targeted modification of the polypeptide backbone. Individual α-residues at solvent-exposed sites throughout the sequence of the GCN4 leucine zipper were replaced by β^3^ or C_α_-Me-α analogues. Although similar in covalent structure, these monomers have dramatically different conformational preferences. Both retain the side chain of the replaced α-residue. Analysis of a representative subset of the GCN4 variants by X-ray crystallography shows they adopt folded structures virtually identical to the prototype coiled coil. The thermodynamics of the folding equilibria for the series vary in complex ways with modification type and context. However, the sensitivity of folding free energy to concentration of added chemical denaturant (the *m* value) is dictated in a predictable manner by the nature of the backbone alteration: α→β^3^ substitution increases *m*, while α→C_α_-Me-α substitution reduces it. The significance of these finding stems from the correlation of the *m* value with differences in solvation between protein folded and unfolded states. Because the folded structures of the regioisomeric coiled coil variants are so similar, the results provide strong support for the hypothesis that targeted backbone modification is exerting a pronounced effect on the structure and dynamics of the unfolded state ensemble. Dual site modification with mutually reinforcing substitutions decreases the change in estimated solvent accessible surface area associated with folding even further. Collectively, the series of coiled coil variants show *m* values ranging from 64% to 120% relative to the prototype. Prior studies on sequence-stability relationships in staphylococcal nuclease showed some single- and dual-site side-chain substitutions can elicit comparable magnitude changes in the *m* value.^62^ What is noteworthy in the present work is that the effects of the two different backbone alterations are predictable based on monomer type and do not involve any change to side-chain functionality.

In considering the origin of the observed effects of backbone modification on the unfolded state ensemble of GCN4-p1, it is useful to reflect on the chemical differences among the backbones under study. Relative to a canonical α-residue, CH_2_ insertion in the backbone from α→β^3^ substitution adds a freely rotatable bond. From first-principles, this would be expected to enhance local conformational freedom and lower propensity for residual secondary structure in that region of the chain in the unfolded state. Supporting this hypothesis, one prior report found the helix propensity of the β^3^ analogue of alanine to be even lower than that of glycine.^63^ With respect to the *m* value, we have showed that variants of the bacterial protein GB1 with four side-chain retaining α→β^3^ replacements in a helical region manifest *m* value increases per substitution comparable to those seen in the present study.^29^ We observed similar qualitative trends in variants of bacterial protein BdpA, but effects in that system were smaller in magnitude and dependent on the substitution context.^28^ In comparison to a canonical α-residue, C_α_-Me-α residues have reduced conformational freedom due to the stereoelectronic effects of geminal C_α_ substitution. The C_α_-Me-α analogue of alanine (aminoisobutyric acid, Aib) is well-known to have a high helical propensity,^64^ greater than that of alanine in a protein context.^65^ However, Aib is achiral so has no preference between a left- or right-handed helical conformation. The presence of a stereocenter in a chiral C_α_-Me-α monomer overcomes this issue and enhances helix propensity even further as a result.^66^ Aib incorporation at four sites in BdpA reduced observed *m* values;^28^ however, similar changes in the miniprotein villin headpiece had little effect.^66^ Future work aimed at direct elucidation of the unfolded state ensemble in these and other systems by experiment and/or simulation will help shed light on the exact molecular impacts of backbone modification on unfolded state dynamics.

Placing the present work in a broader context, an important feature of the backbone engineering scheme described here is that it is complementary to a wide range of approaches previously utilized to study the role of the unfolded state in the folding process. Side chain replacement by mutagenesis has been and remains a premiere method for engineering protein properties, including the unfolded state. Because the backbone modifications retain the side chain of each replaced α-residue, side-chain sequence and backbone composition are orthogonal and can be independently varied. The same holds for characterization methods. Here, we used the *m* value as a readily accessible metric to test the hypothesis of altered unfolded state conformational properties. The possibility exists to apply advanced experimental biophysical methods as well as simulation in conjunction with backbone engineering to obtain higher resolution structural information about the unfolded ensemble. Finally, application of the scheme described here in conjunction with analysis of folding kinetics has the potential to provide a new avenue to study protein folding mechanisms by modulating conformational properties of the transition state ensemble. In terms of generalizability, the approach described here is potentially applicable to a wide range of systems. However, an important limitation is that modifications are restricted to sites that are helical in the folded state. Further, the protein to be studied must be accessible by total chemical synthesis or semisynthesis to introduce the chemical modifications. These limitations notwithstanding, it is our hope that the approach described here will serve as a useful addition to the experimental toolbox for fundamental studies of the folding physics of proteins and protein mimetics. Efforts to explore its application to this end are ongoing in our laboratory.

## Supplementary Material



## Notes

HPLC and MS characterization data for purified peptides; HPLC data related to the side-reaction in the synthesis of **α^Me^S14**; CD data from thermal and chemical denaturation experiments; details on buffers used for crystallization and cryoprotection in X-ray crystallography experiments; crystallographic data collection and refinement statistics for new reported crystal structures (PDF)
