# Supplementary material for: Manipulating the Unfolded State of a Folded Protein through Site-Specific Backbone Modification
Source: Biochemistry. 2026 Mar 3;65(6):693–703. doi: 10.1021/acs.biochem.5c00687 (PMC13001086; doi:10.1021/acs.biochem.5c00687)
Supplement: Supplementary file 1 [file bi5c00687_si_001.pdf]

# Manipulating the Unfolded State of a Folded Protein through Site-specific Backbone Modification

Gabrielle E. Page, Yuhan Lin, W. Seth Horne*

*Department of Chemistry, University of Pittsburgh, Pittsburgh, PA 15260 (USA)*

horne@pitt.edu

**SUPPORTING INFORMATION**

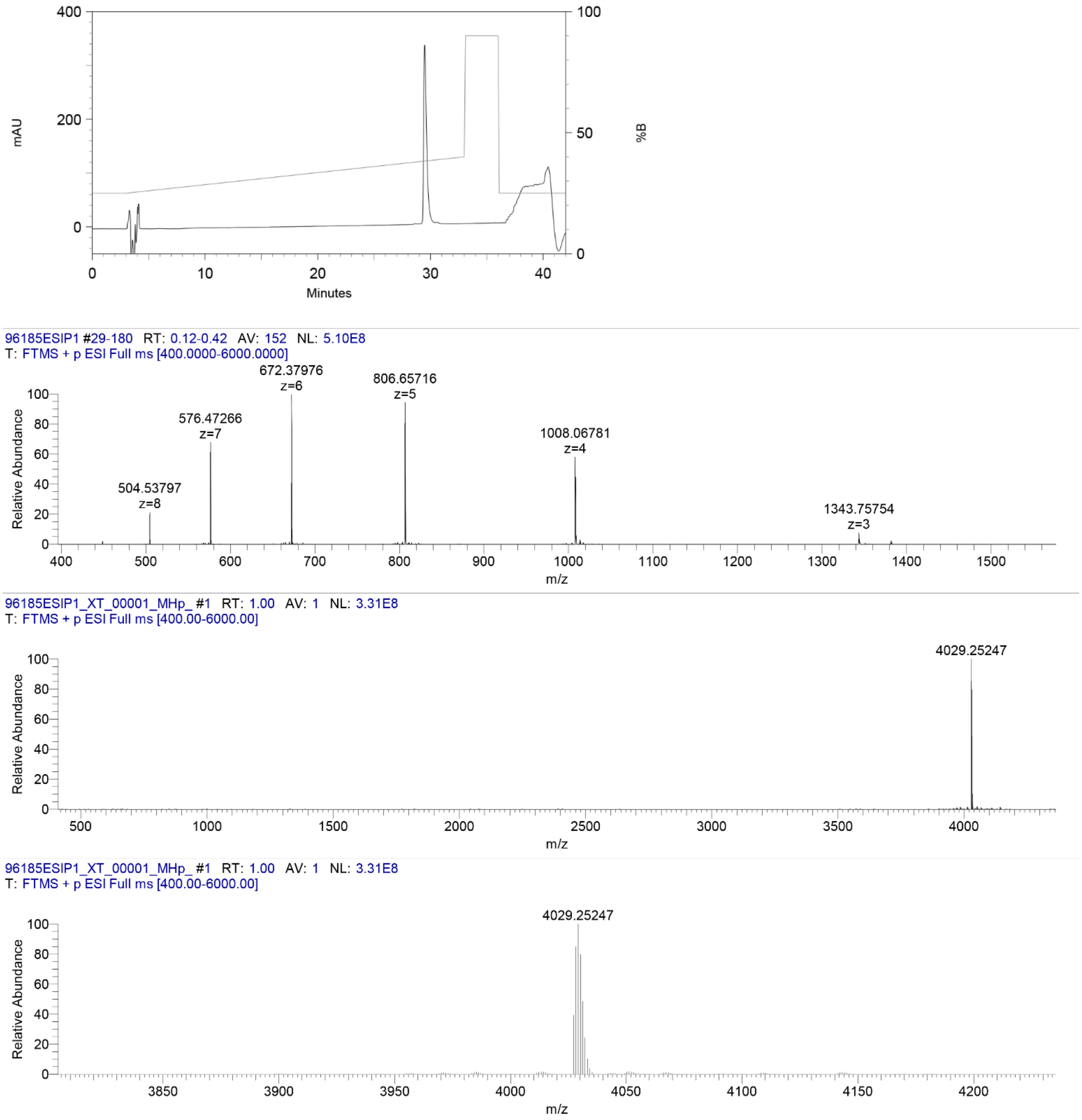


**Figure S1.** Analytical HPLC (25-40% solvent B over 30 min) and ESI-MS data (top: raw spectrum; middle: deconvoluted spectrum; bottom: zoomed view of deconvoluted spectrum) for purified **p1**. $[M+H]^+$ calc. is 4029.26 Da for the most abundant isotope.

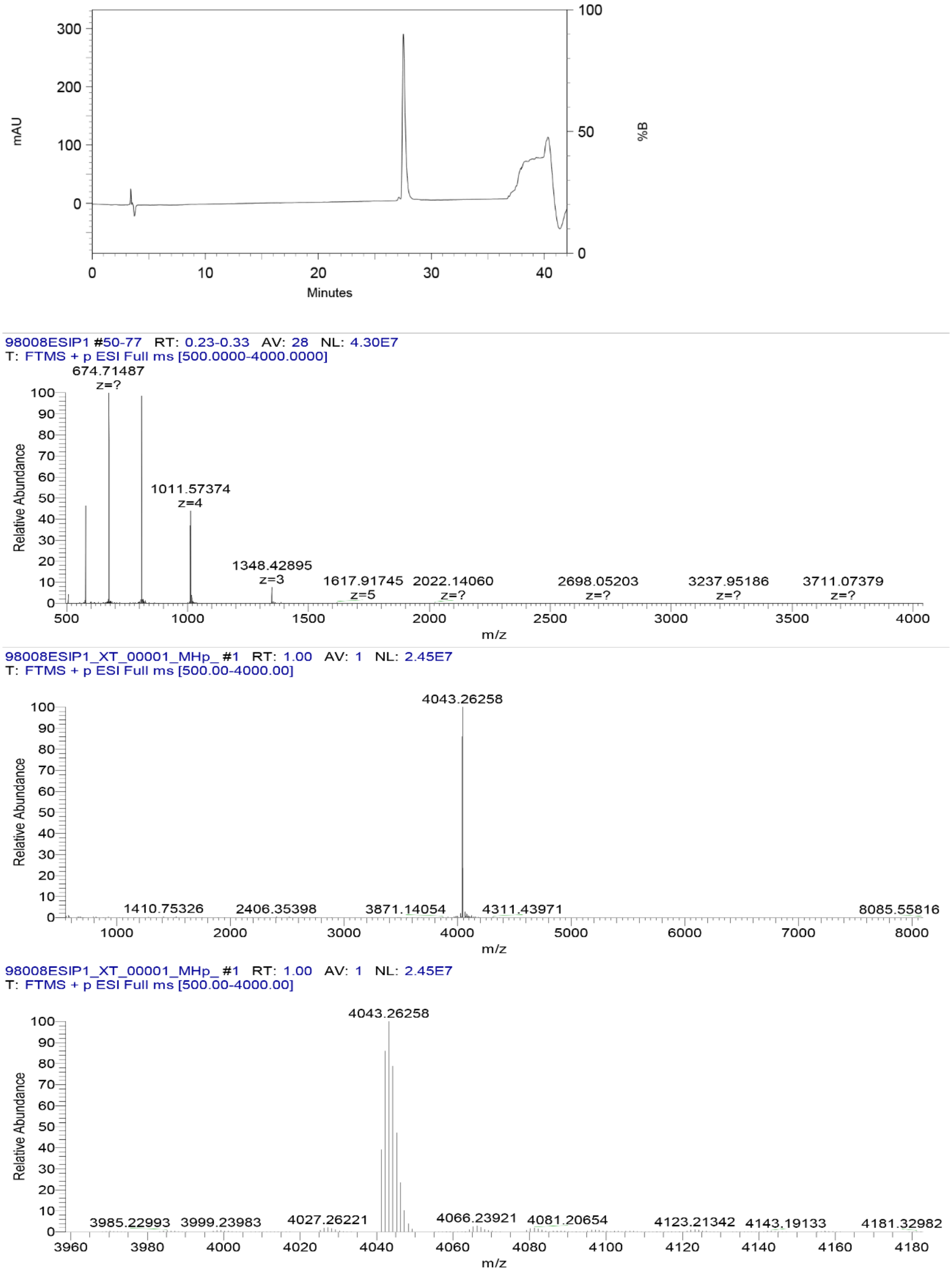


**Figure S2.** Analytical HPLC (25-40% solvent B over 30 min) and ESI-MS data (top: raw spectrum; middle: deconvoluted spectrum; bottom: zoomed view of deconvoluted spectrum) for purified **β$^3$D7**. $[M+H]^+$ calc. is 4043.27 Da for the most abundant isotope.

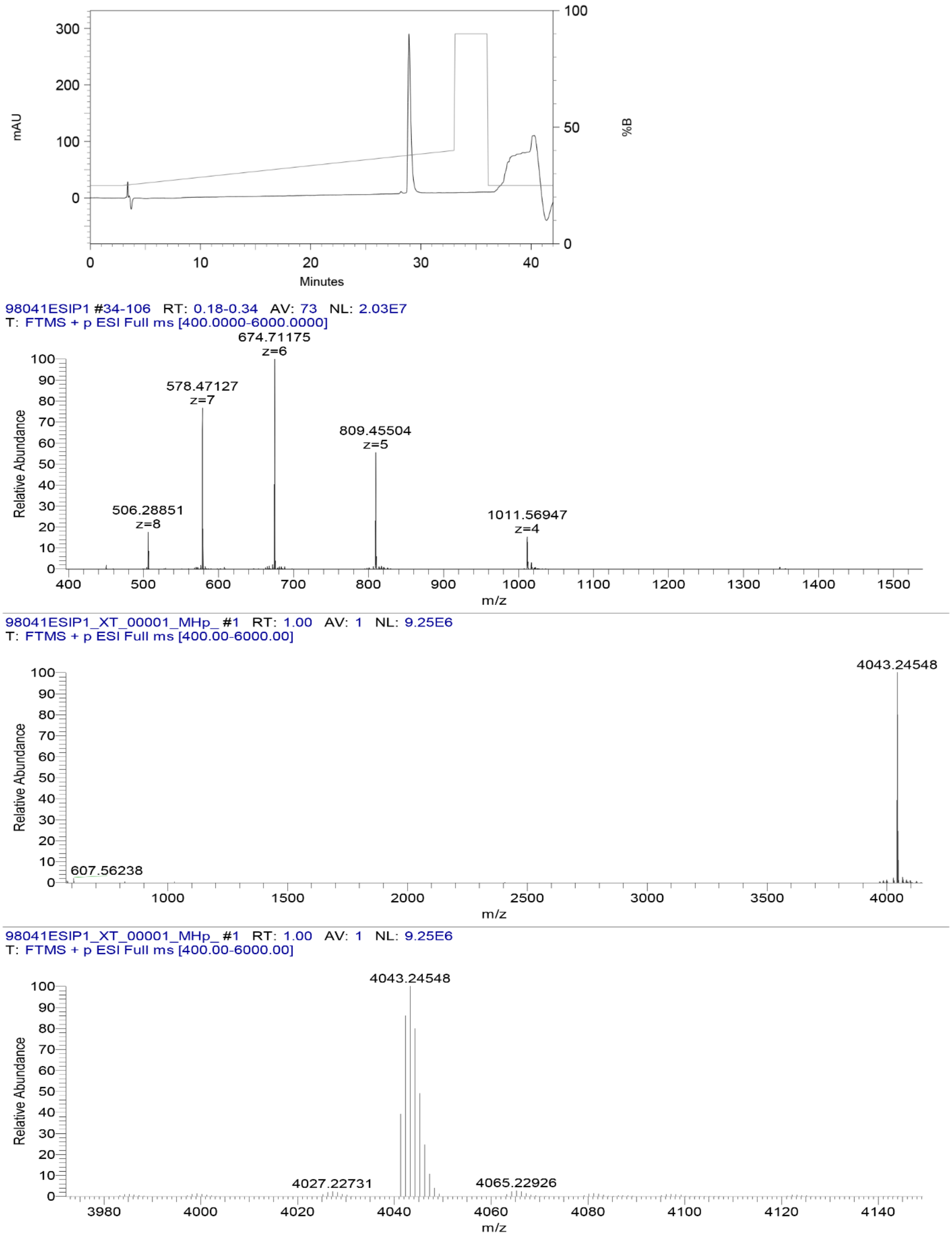


**Figure S3.** Analytical HPLC (25-40% solvent B over 30 min) and ESI-MS data (top: raw spectrum; middle: deconvoluted spectrum; bottom: zoomed view of deconvoluted spectrum) for purified **α$^{Me}$D7**. [M+H]$^+$ calc. is 4043.27 Da for the most abundant isotope.

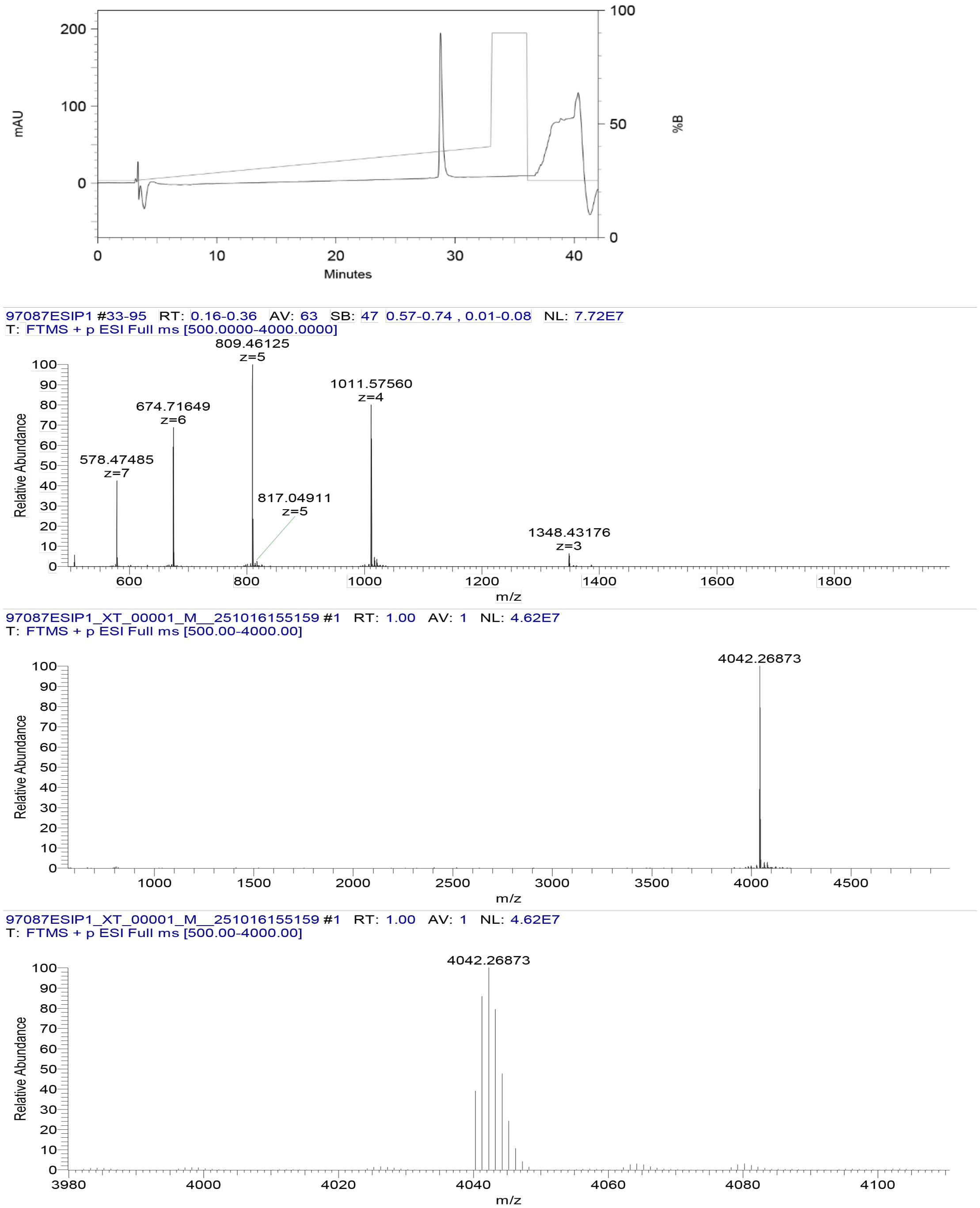


**Figure S4.** Analytical HPLC (25-40% solvent B over 30 min) and ESI-MS data (top: raw spectrum; middle: deconvoluted spectrum; bottom: zoomed view of deconvoluted spectrum) for purified **$\beta^3$E11**. $[M+H]^+$ calc. is 4043.27 Da for the most abundant isotope.

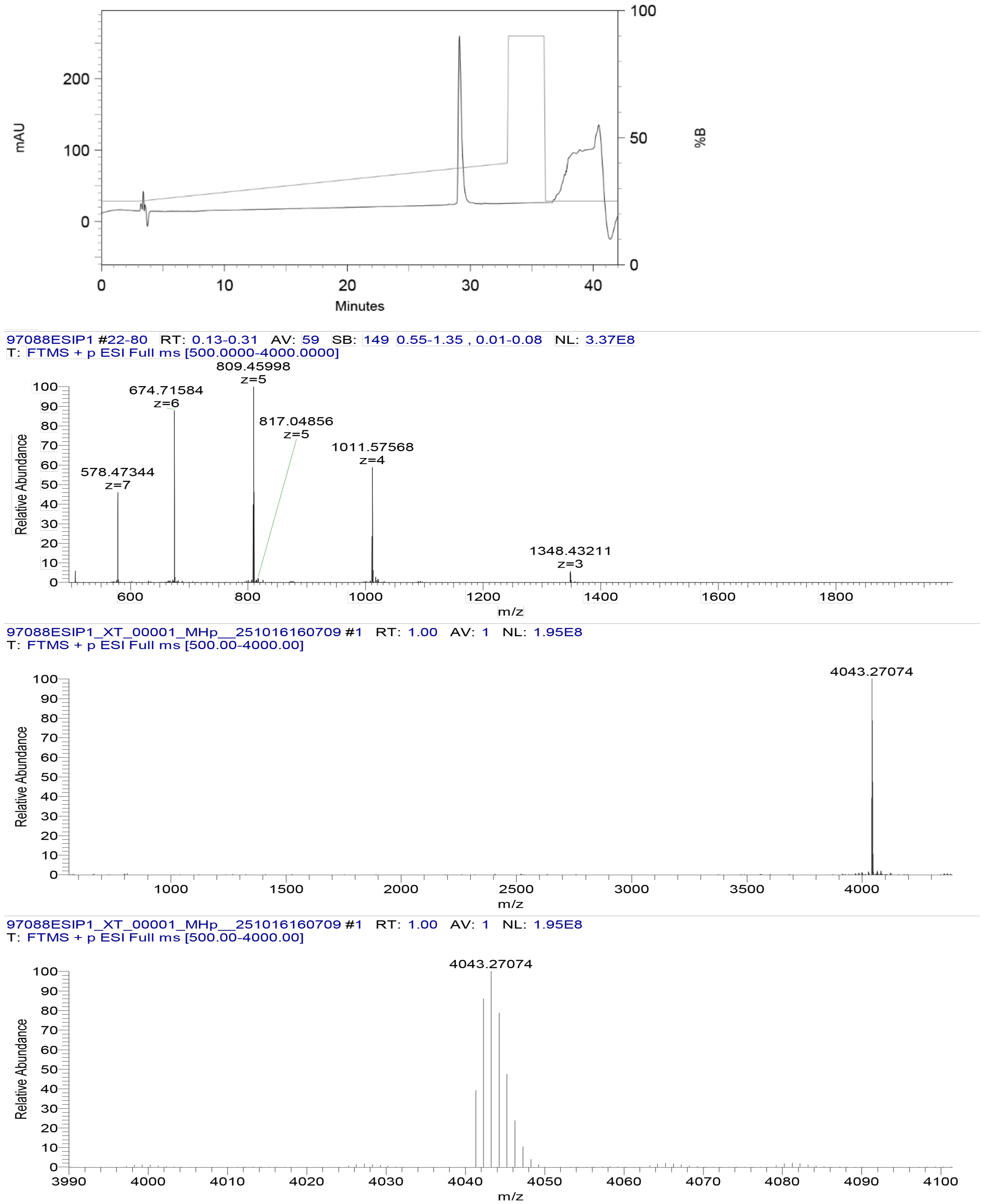


**Figure S5.** Analytical HPLC (25-40% solvent B over 30 min) and ESI-MS data (top: raw spectrum; middle: deconvoluted spectrum; bottom: zoomed view of deconvoluted spectrum) for purified $\alpha^{Me}$**E11**. $[M+H]^+$ calc. is 4043.27 Da for the most abundant isotope.

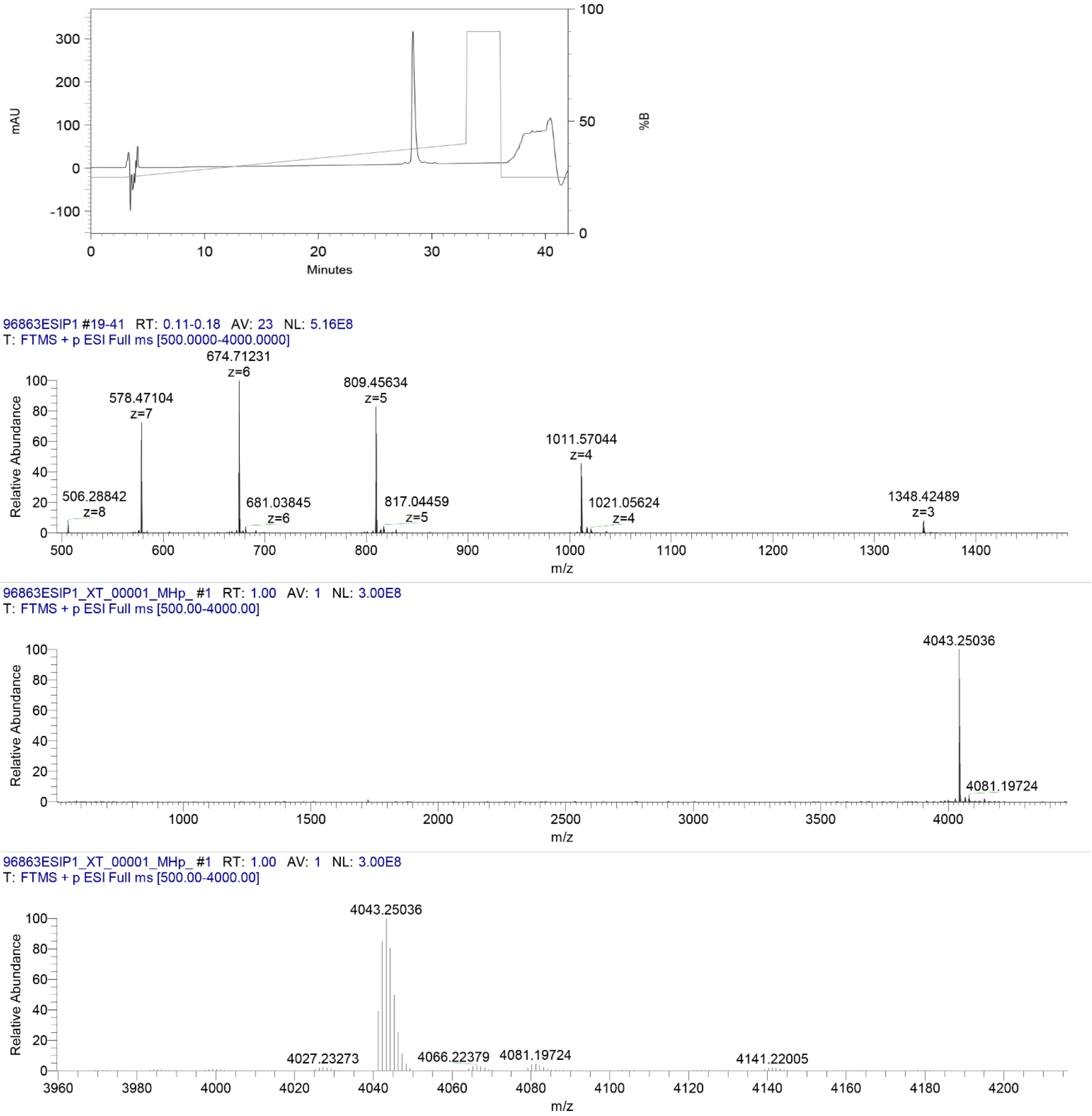


**Figure S6.** Analytical HPLC (25-40% solvent B over 30 min) and ESI-MS data (top: raw spectrum; middle: deconvoluted spectrum; bottom: zoomed view of deconvoluted spectrum) for purified **β$^3$S14**. $[M+H]^+$ calc. is 4043.27 Da for the most abundant isotope.

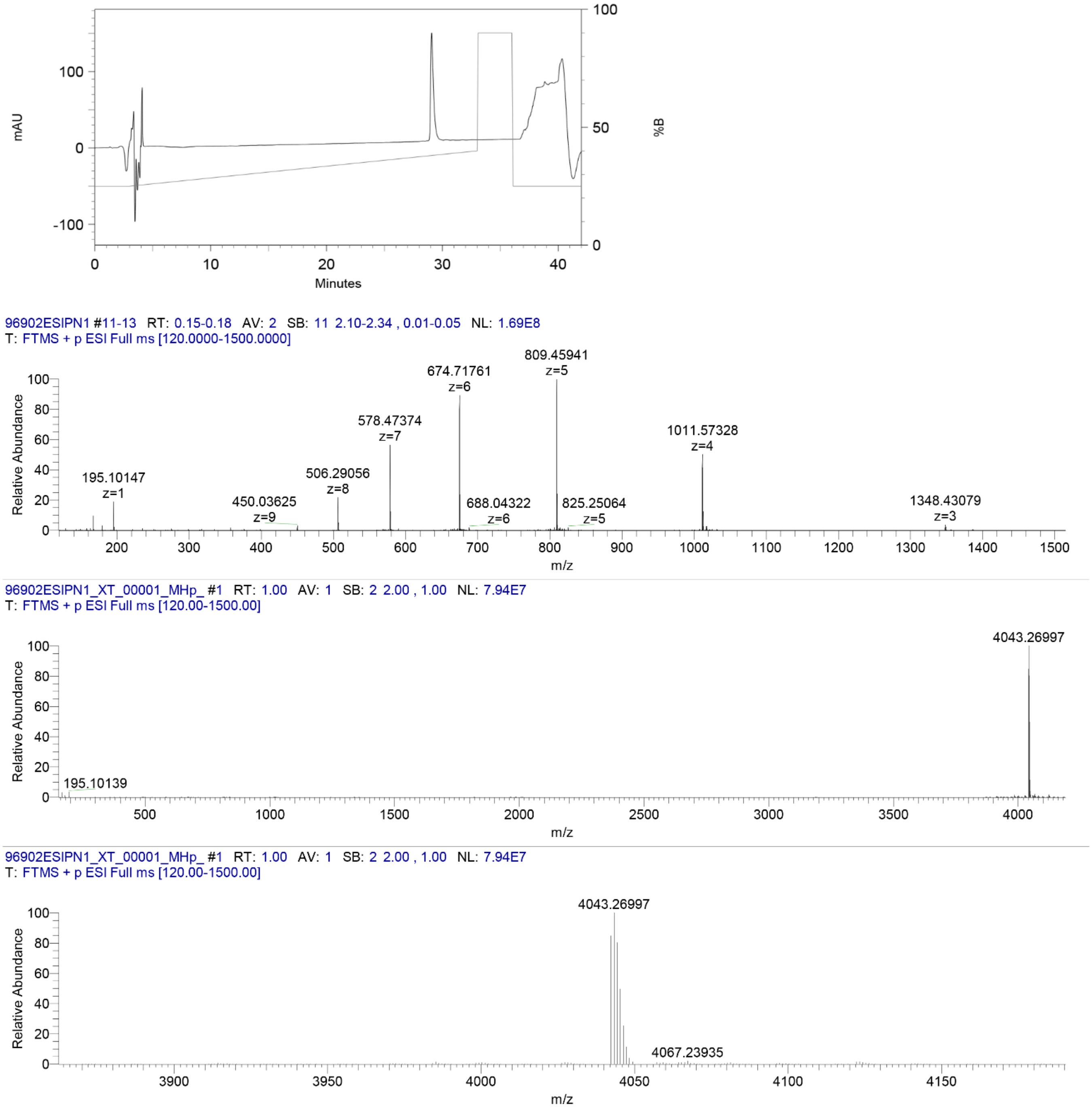


**Figure S7.** Analytical HPLC (25-40% solvent B over 30 min) and ESI-MS data (top: raw spectrum; middle: deconvoluted spectrum; bottom: zoomed view of deconvoluted spectrum) for purified **α$^{Me}$S14**. $[M+H]^+$ calc. is 4043.27 Da for the most abundant isotope.

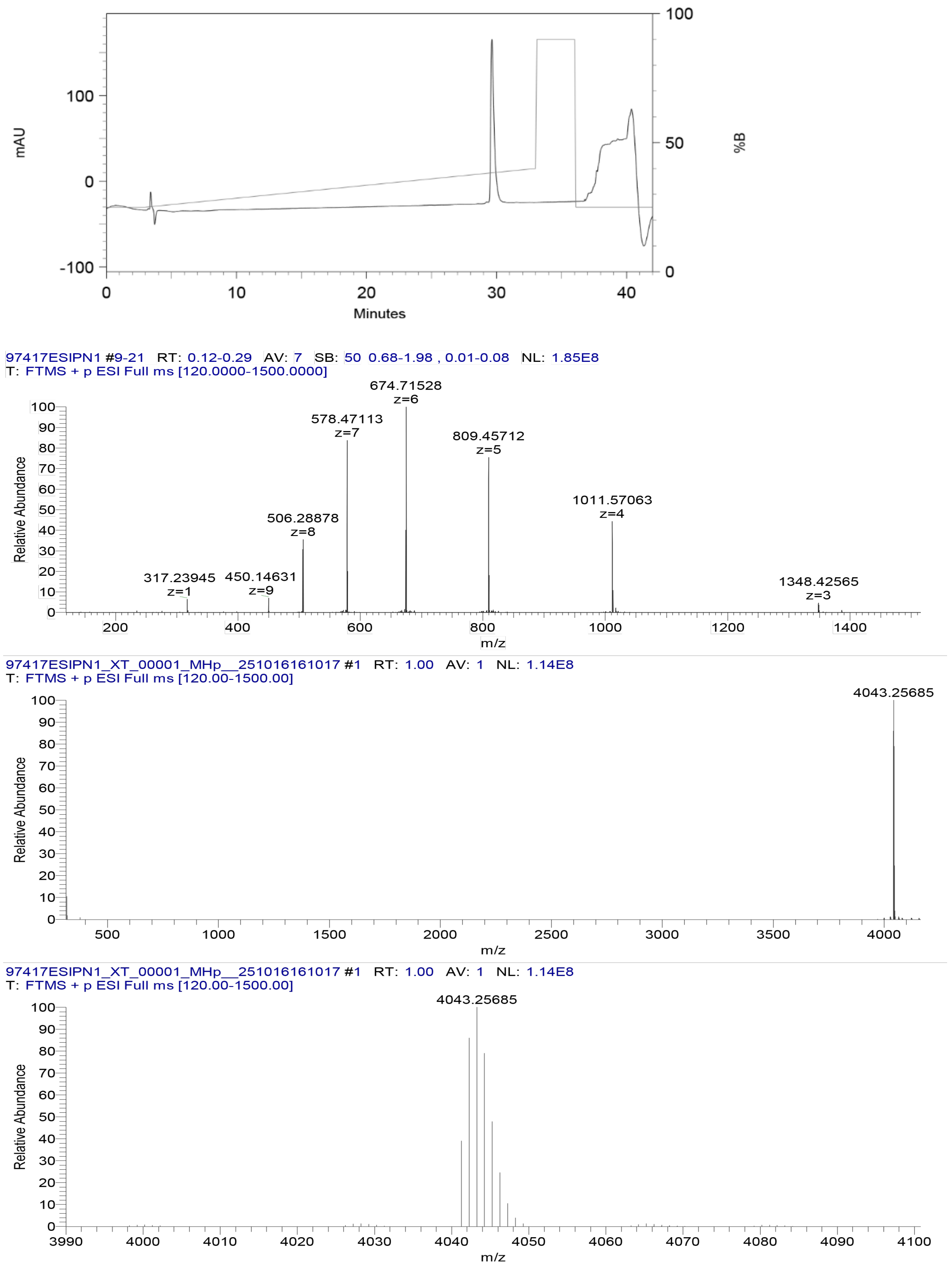


**Figure S8.** Analytical HPLC (25-40% solvent B over 30 min) and ESI-MS data (top: raw spectrum; middle: deconvoluted spectrum; bottom: zoomed view of deconvoluted spectrum) for purified **β$^3$E20**. $[M+H]^+$ calc. is 4043.27 Da for the most abundant isotope.

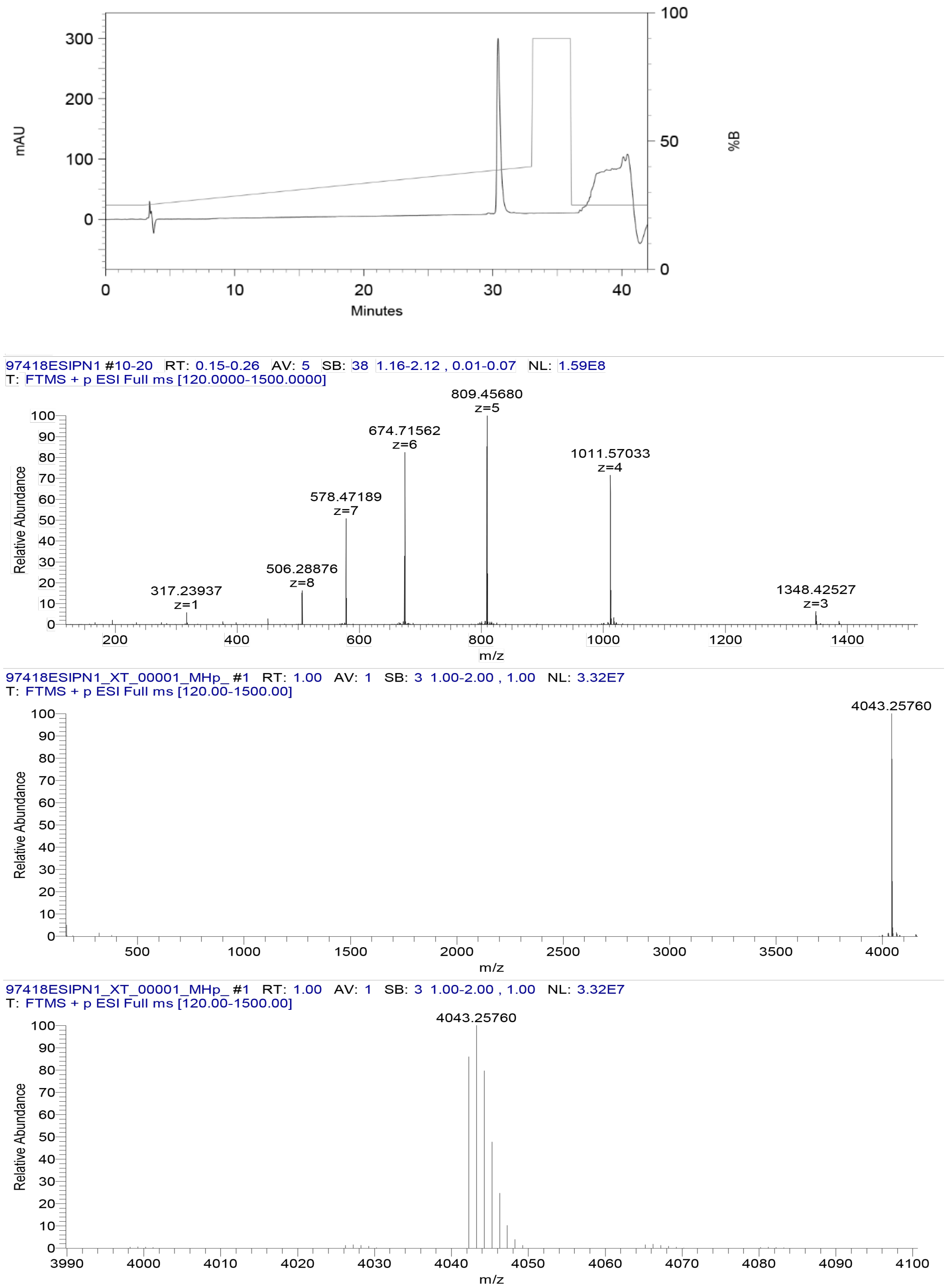


**Figure S9.** Analytical HPLC (25-40% solvent B over 30 min) and ESI-MS data (top: raw spectrum; middle: deconvoluted spectrum; bottom: zoomed view of deconvoluted spectrum) for purified **α$^{Me}$E20**. $[M+H]^+$ calc. is 4043.27 Da for the most abundant isotope.

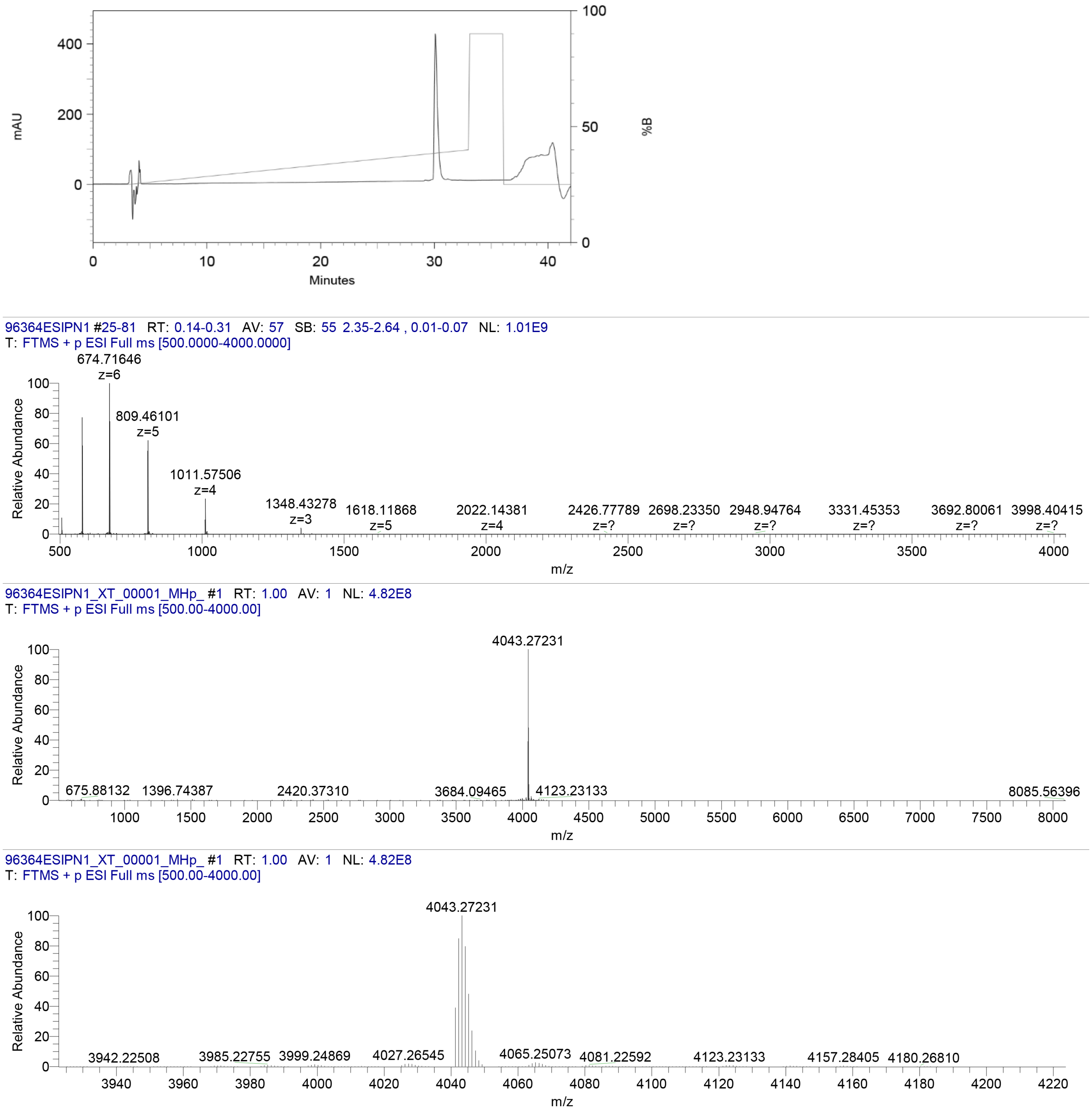


**Figure S10.** Analytical HPLC (25-40% solvent B over 30 min) and ESI-MS data (top: raw spectrum; middle: deconvoluted spectrum; bottom: zoomed view of deconvoluted spectrum) for purified **β$^3$A24**. $[M+H]^+$ calc. is 4043.27 Da for the most abundant isotope.

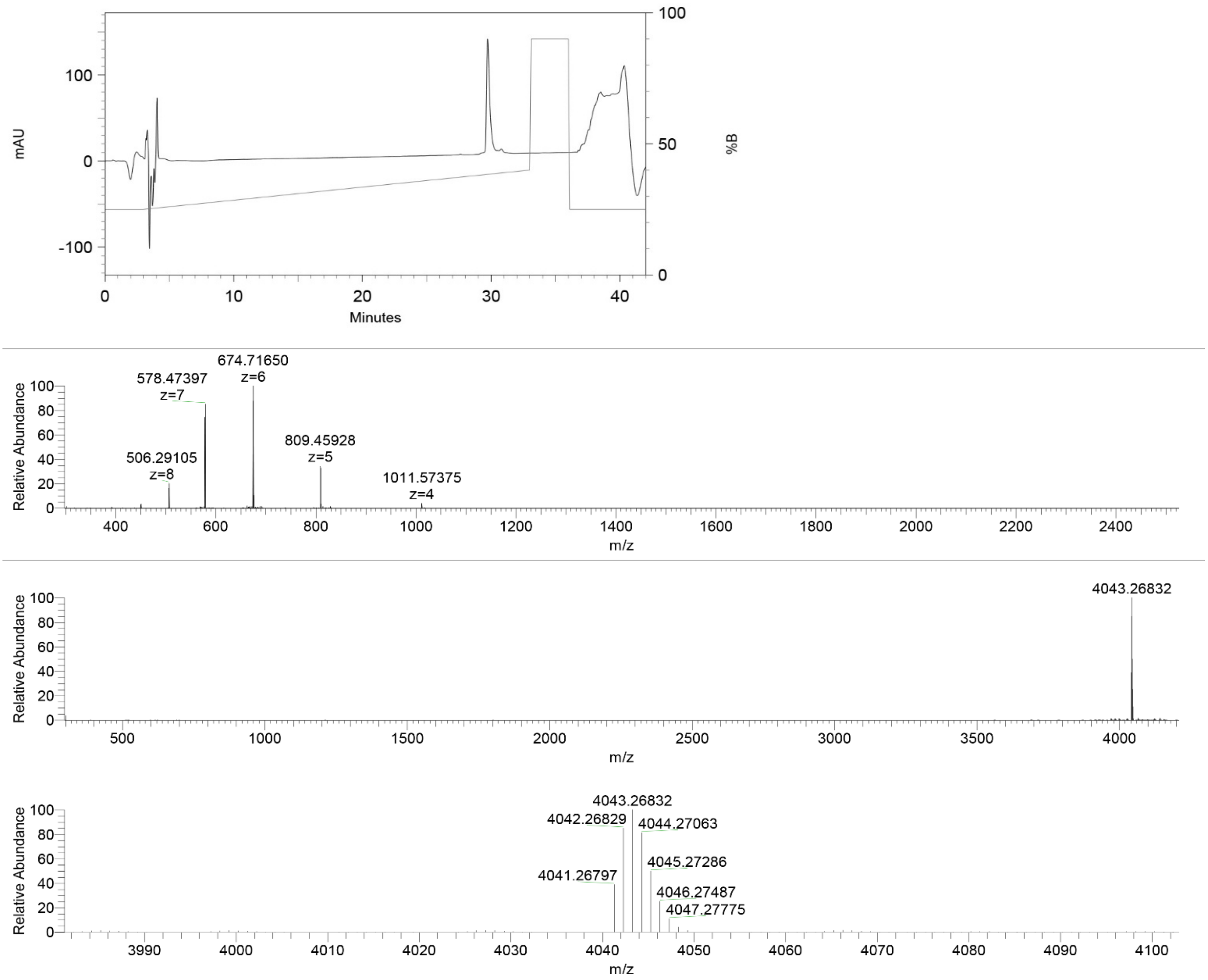


**Figure S11.** Analytical HPLC (25-40% solvent B over 30 min) and ESI-MS data (top: raw spectrum; middle: deconvoluted spectrum; bottom: zoomed view of deconvoluted spectrum) for purified **$\alpha^{Me}$A24**. $[M+H]^+$ calc. is 4043.27 Da for the most abundant isotope.

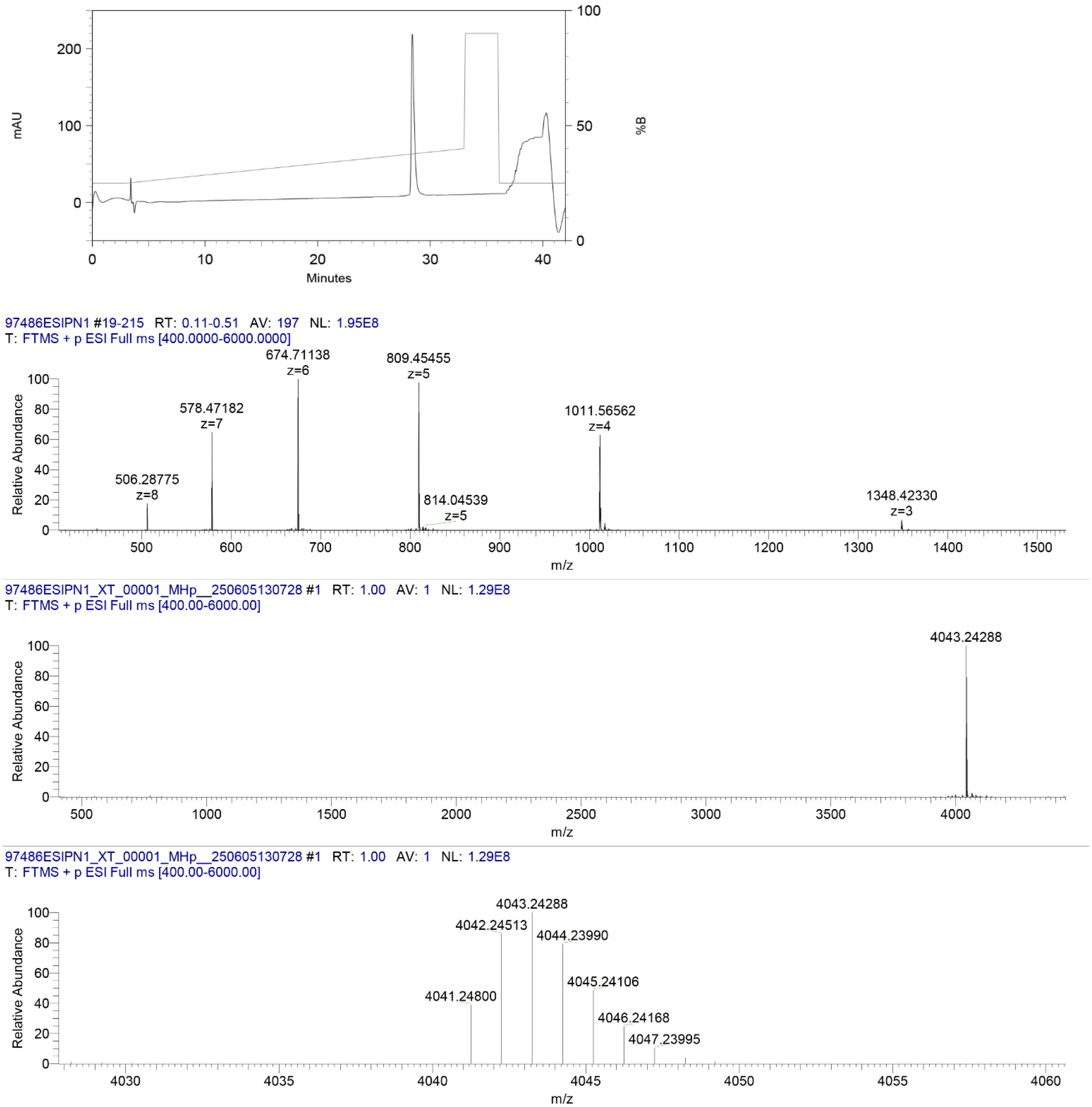


**Figure S12.** Analytical HPLC (25-40% solvent B over 30 min) and ESI-MS data (top: raw spectrum; middle: deconvoluted spectrum; bottom: zoomed view of deconvoluted spectrum) for purified **$\beta^3$K28**. $[M+H]^+$ calc. is 4043.27 Da for the most abundant isotope.

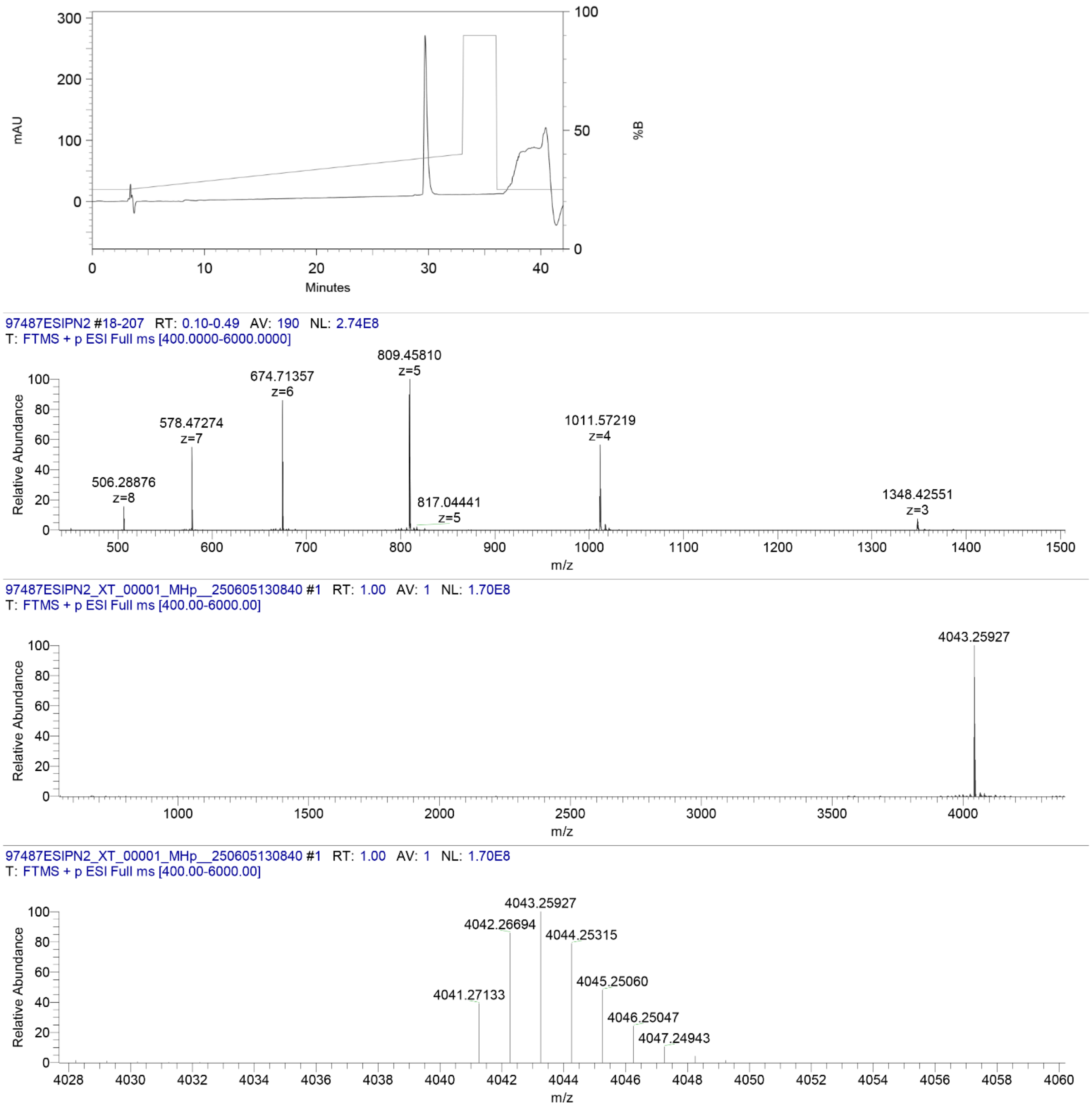


**Figure S13.** Analytical HPLC (25-40% solvent B over 30 min) and ESI-MS data (top: raw spectrum; middle: deconvoluted spectrum; bottom: zoomed view of deconvoluted spectrum) for purified **α$^{\mathbf{Me}}$K28**. $[M+H]^+$ calc. is 4043.27 Da for the most abundant isotope.

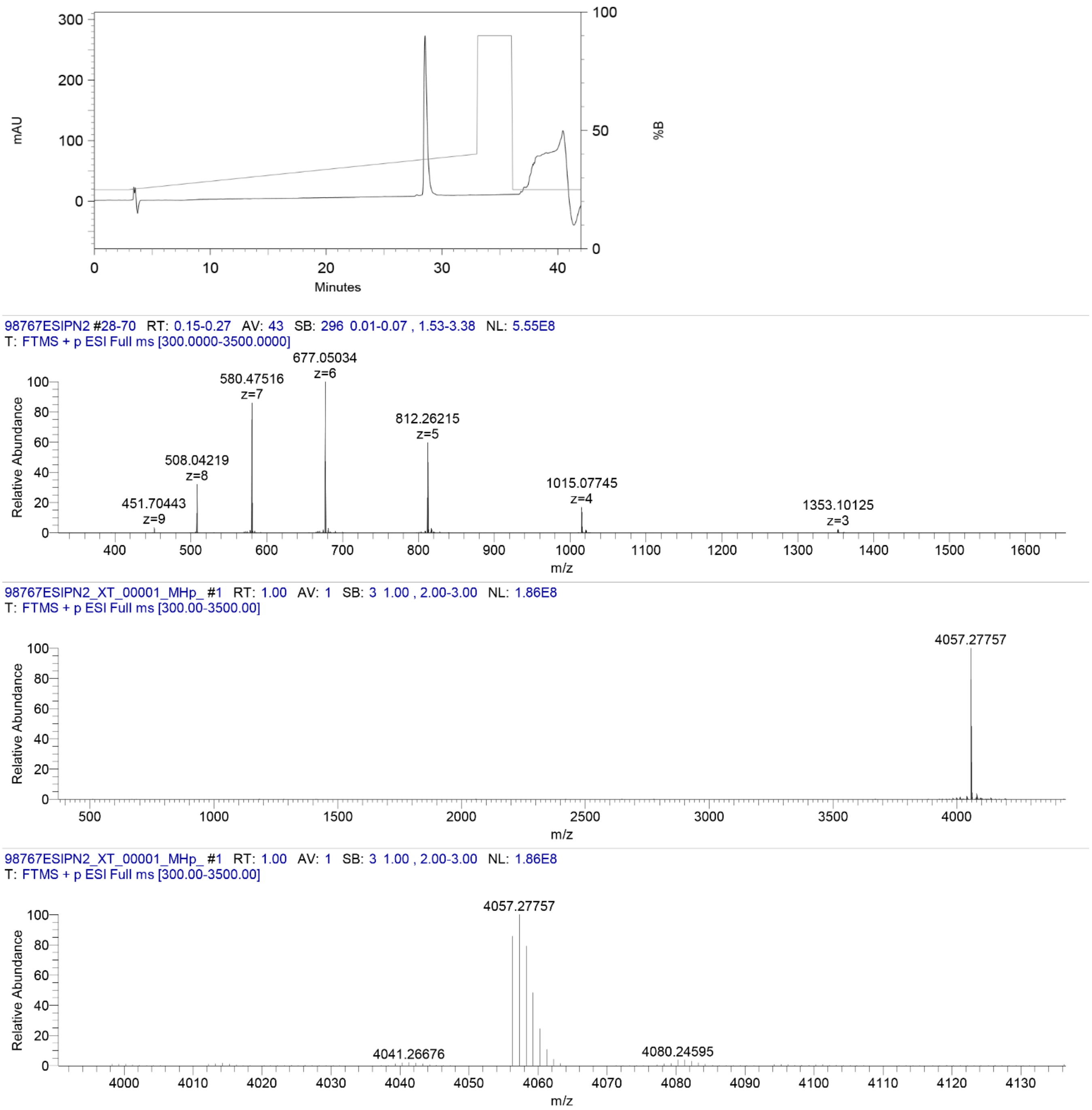


**Figure S14.** Analytical HPLC (25-40% solvent B over 30 min) and ESI-MS data (top: raw spectrum; middle: deconvoluted spectrum; bottom: zoomed view of deconvoluted spectrum) for purified **α<sup>Me</sup>D7/ α<sup>Me</sup>K28**. $[M+H]^+$ calc. is 4057.29 Da for the most abundant isotope.

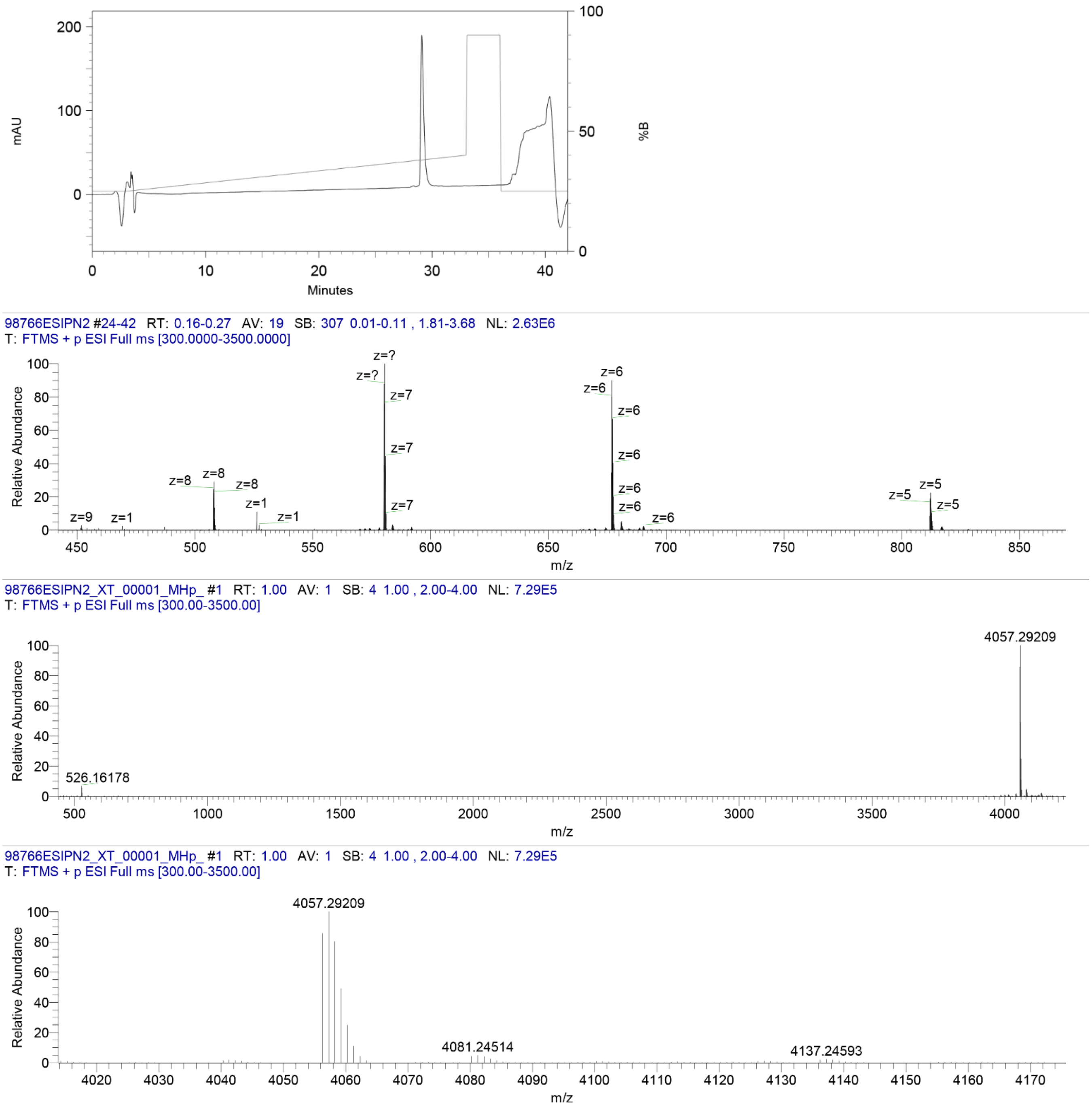


**Figure S15.** Analytical HPLC (25-40% solvent B over 30 min) and ESI-MS data (top: raw spectrum; middle: deconvoluted spectrum; bottom: zoomed view of deconvoluted spectrum) for purified **α$^{Me}$E11/ α$^{Me}$S14**. $[M+H]^+$ calc. is 4057.29 Da for the most abundant isotope.

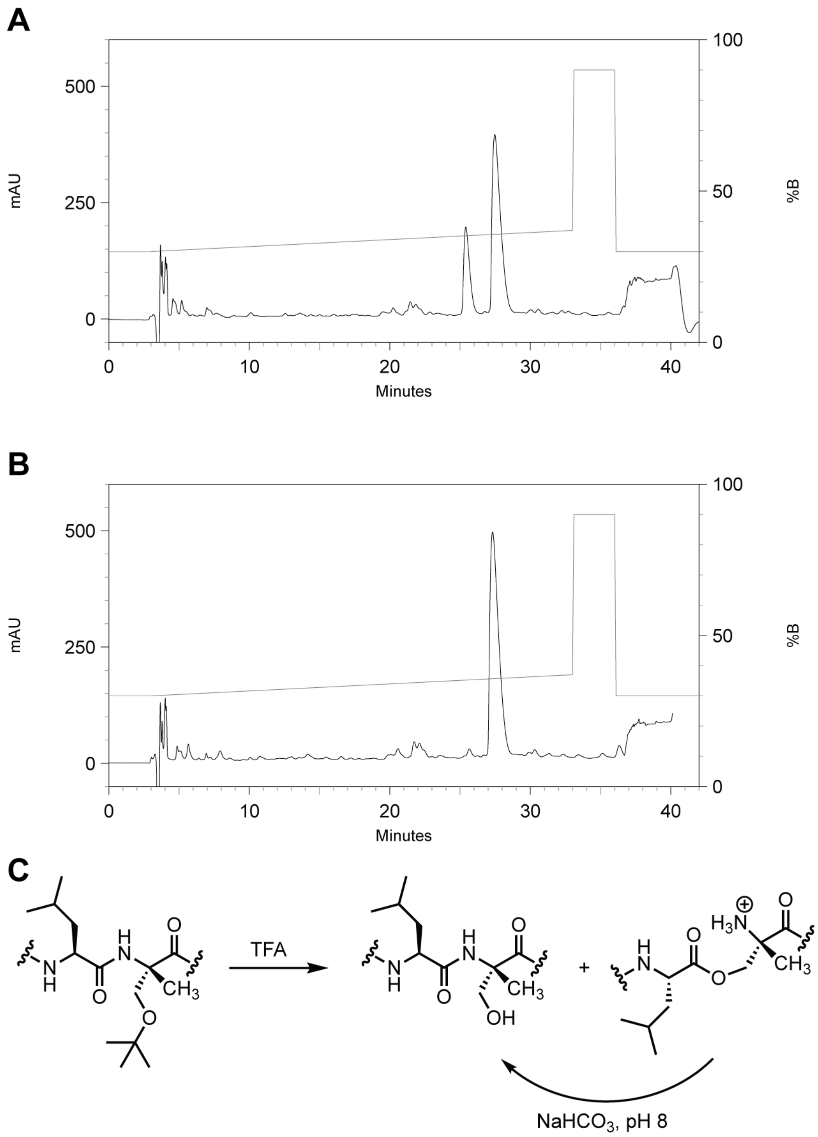


**Figure S16.** Characterization of a side reaction observed in the synthesis of variant **α$^{Me}$S14**. (A) Analytical HPLC of the crude mixture obtained after TFA cleavage (30-37% solvent B over 30 min). Two major peaks are observed in a ~3:1 ratio, both with mass corresponding to the expected product. The peak ratio did not change on extended sonication, and the two species could be isolated by preparative HPLC and and lyophilized without interconversion. (B) Analytical HPLC chromatogram of material from (A) after exposure to 60 mM bicarbonate at pH ~8 overnight. The minor peak is consumed with a corresponding increase in the major peak. (C) Putative chemical process giving rise to the above observations. Partial O→N acyl migration at the αMeSer during TFA cleavage gives a mixture of peptide and depsipeptide. Subjecting this mixture to mildly basic conditions leads to quantitative conversion of the depsipeptide to the desired N-acyl product. The same behavior noted above was also observed for double variant **α$^{Me}$E11/α$^{Me}$S14** but not any other variant from the present study (data not shown). This suggests the side-reaction is specific to αMeSer.

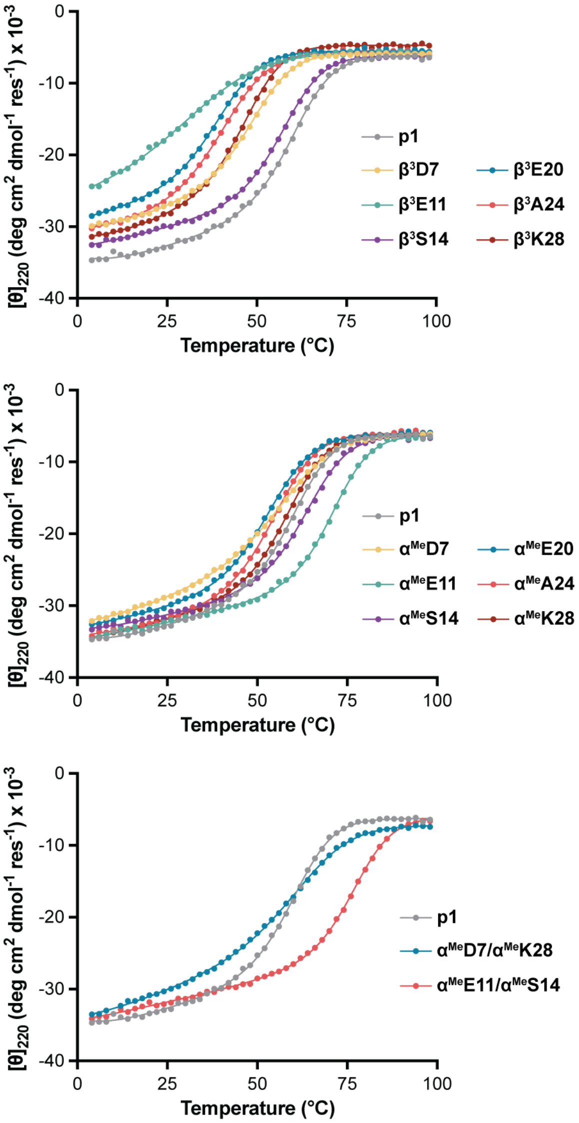


**Figure S17.** Circular dichroism thermal melts for **p1** and variants. Conditions: 50 µM peptide in 20 mM phosphate, pH 7 at 20°C. Points depict observed molar ellipticity and lines show fits to a two-state folding model.

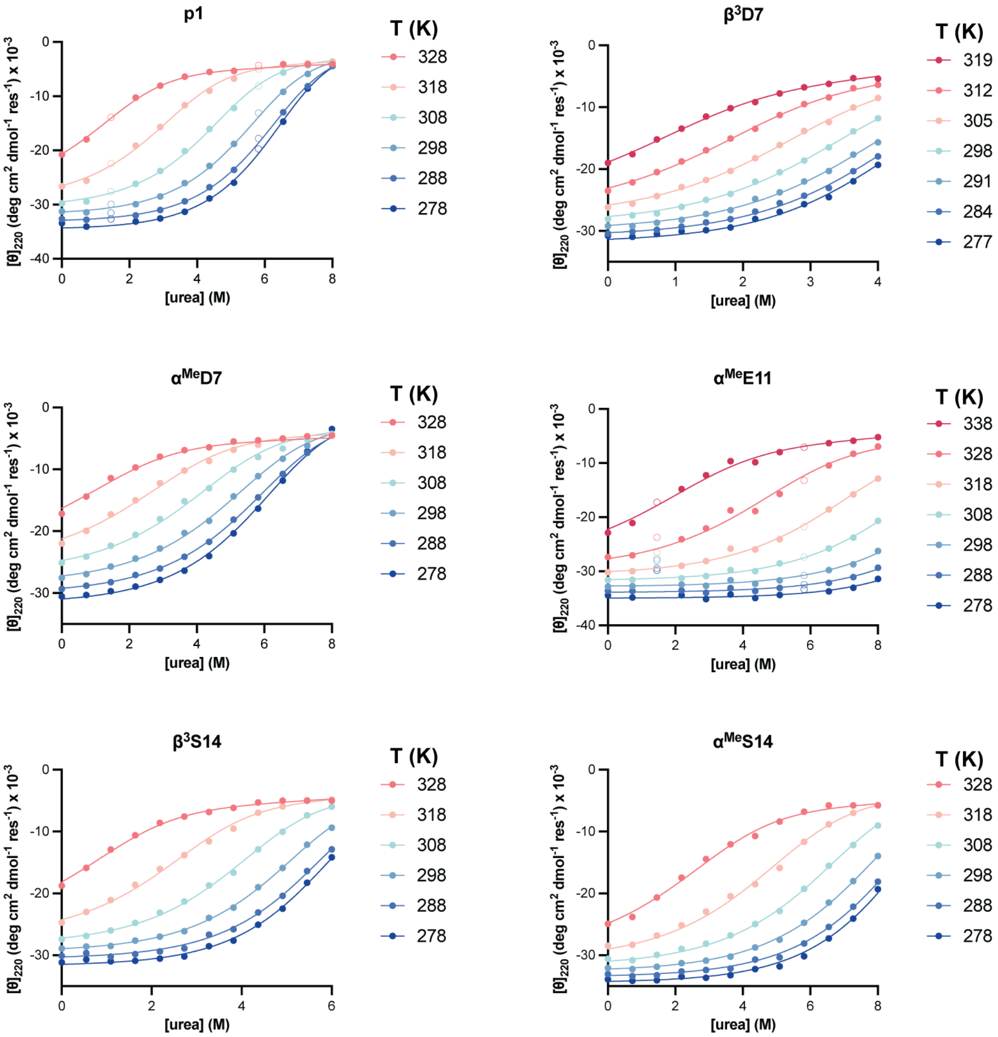


**Figure S18.** Thermal / chemical denaturation monitored by circular dichroism for **p1** and variants. Points are experimentally observed molar ellipticity for a sample of 50 µM peptide in 20 mM phosphate pH 7 at the indicated temperature and urea concentration. Lines depict a global fit of the data set to a two-state monomer-dimer folding equilibrium. For a subset of experiments, one of the six positions in the CD sample changer gave aberrant results from the remainder of the data set. Measurements from these samples (shown as open circles) were excluded from the fits.

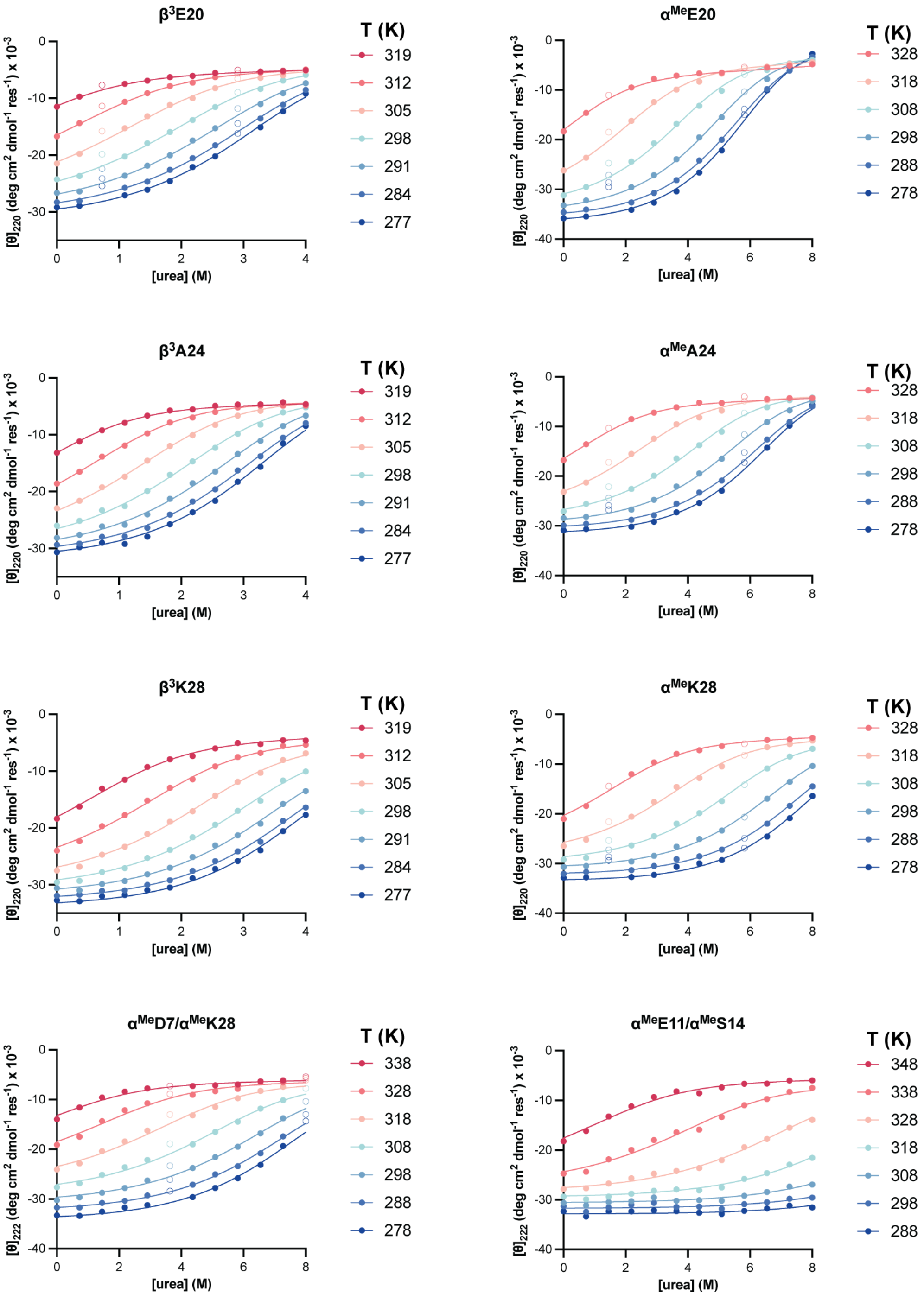


**Figure S19.** Thermal / chemical denaturation monitored by circular dichroism for **p1** variants. Points are experimentally observed molar ellipticity for a sample of 50 μM peptide in 20 mM phosphate pH 7 at the indicated temperature and urea concentration. Lines depict a global fit of the data set to a two-state monomer-dimer folding equilibrium. For a subset of experiments, one of the six positions in the CD sample changer gave aberrant results from the remainder of the data set. Data from these samples (shown as open circles) were excluded from the fits.

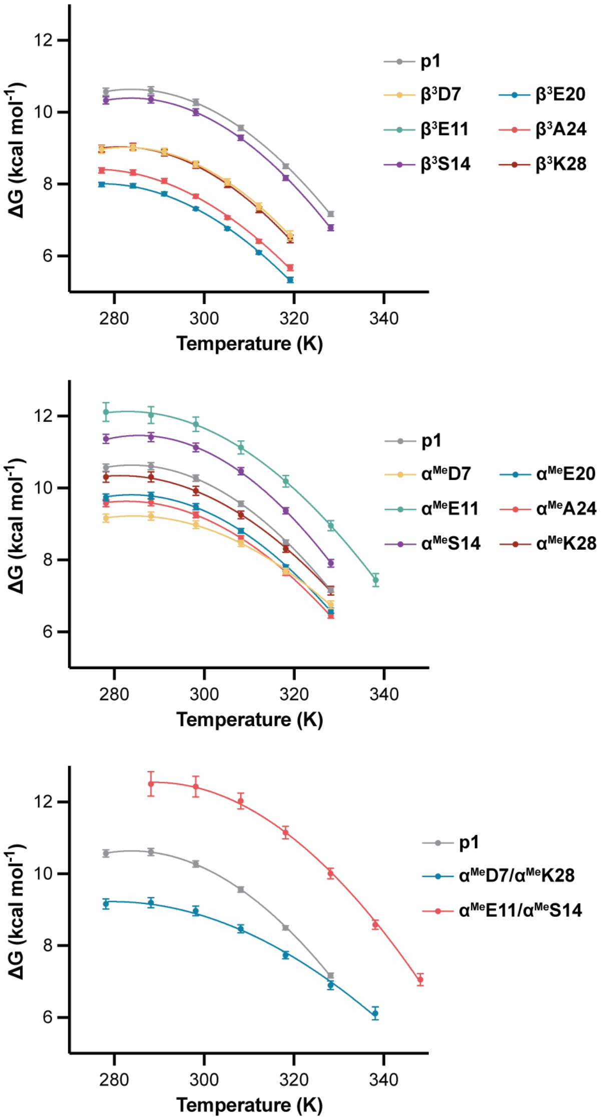


**Figure S20.** Free energy of unfolding (ΔG) as a function of temperature for **p1** and variants. Data points are determined from fits in Figures S18-S19 and error bars the parameter uncertainties from the fits. The line depicts a fit of data for each variant to the Gibbs Helmholtz equation, which yields the enthalpy (ΔH°), entropy (ΔS°), and heat capacity change ($\Delta C_p$) associated with unfolding.

**Table S1.** Buffers used for crystallization and cryoprotection of **p1** and variants for X-ray diffraction experiments.

| **Peptide** | **Crystallization buffer**[a] | **Cryoprotection buffer** |
|---|---|---|
| **p1** | 0.2 M sodium acetate pH 4.5, 0.1 M sodium citrate pH 5.6, 10% w/v PEG 3350 | Crystallization buffer with 10% v/v glycerol |
| **$\alpha^{Me}$E11** | 0.3 M sodium acetate pH 4.6, 0.1 M sodium citrate pH 4.6, 5% w/v PEG 3350 | Crystallization buffer with 15% v/v glycerol |
| **$\beta^{3}$E20** | 0.3 M sodium acetate pH 4.6, 0.1 M sodium citrate pH 4.6, 5% w/v PEG 3350 | Crystallization buffer with 10% v/v glycerol |
| **$\beta^{3}$A24** | 0.2 M sodium acetate pH 4.6, 0.1 M sodium citrate pH 4.6, 10% w/v PEG 3350 | Crystallization buffer with 10% v/v glycerol |
| **$\alpha^{Me}$A24** | 0.2 M sodium acetate pH 4.6, 0.1 M sodium citrate pH 4.6, 10% w/v PEG 3350 | Crystallization buffer with 10% v/v glycerol |
| **$\beta^{3}$K28** | 0.2 M sodium acetate pH 4.6, 0.1 M sodium citrate pH 4.6, 10% w/v PEG 3350 | Crystallization buffer with 15% v/v glycerol |
| **$\alpha^{Me}$K28** | 0.2 M sodium acetate pH 4.6, 0.1 M sodium citrate pH 4.6, 15% w/v PEG 3350 | Crystallization buffer with 15% v/v glycerol |

[a] Crystallization buffers were prepared from 1 M stocks of sodium acetate and sodium citrate at the indicated pH and were not pH adjusted after mixing.

**Table S2.** Crystallographic data collection and refinement statistics for **p1**, **$\alpha^{Me}$E11**, **$\beta^3$E20**, and **$\beta^3$A24**.

| | **p1** | **$\alpha^{Me}$E11** | **$\beta^3$E20** | **$\beta^3$A24** |
|---|---|---|---|---|
| **PDB** | 9Z1P | 9Z1Q | 9Z1R | 9Z1S |
| **Data Collection** | | | | |
| Unit cell dimensions (Å, °) | $a$ = 78.2, $b$ = 30.4, $c$ = 27.8, $\alpha$ = $\gamma$ = 90, $\beta$ = 96.9 | $a$ = 79.2, $b$ = 30.6, $c$ = 27.4 $\alpha$ = $\gamma$ = 90, $\beta$ = 96.4 | $a$ = 27.7, $b$ = 30.6, $c$ = 43.8, $\alpha$ = 103.3, $\beta$ = 102.7, $\gamma$ = 90.1 | $a$ = 84.3, $b$ = 30.9, $c$ = 27.9, $\alpha$ = $\gamma$ = 90, $\beta$ = 102.0 |
| Space group | C2 | C2 | P1 | C2 |
| Resolution (Å) | 50.00–1.60 (1.70–1.60) | 50.00–1.80 (1.91–1.80) | 50.00–1.90 (2.02–1.90) | 50.00–1.75 (1.86–1.75) |
| Total observations | 54,862 | 21,567 | 43,653 | 47,914 |
| Unique observations | 8,725 | 6,143 | 10,637 | 7,123 |
| Redundancy | 6.3 (4.5) | 3.5 (3.4) | 4.1 (3.9) | 6.7 (5.6) |
| Completeness (%) | 99.8 (99.6) | 99.5 (98.4) | 99.0 (96.4) | 98.3 (96.6) |
| $I/\sigma$ | 26.1 (3.5) | 18.8 (4.3) | 16.6 (5.9) | 30.3 (4.1) |
| $R_{meas}$ | 0.053 (0.442) | 0.059 (0.298) | 0.070 (0.225) | 0.051 (0.476) |
| **Refinement** | | | | |
| Resolution (Å) | 23.91-1.60 | 28.49–1.80 | 41.48-1.90 | 28.9-1.75 |
| $R$ | 0.188 | 0.1759 | 0.176 | 0.208 |
| $R_{free}$ | 0.230 | 0.1956 | 0.214 | 0.233 |
| Avg. B factor (Å$^2$) | 17.8 | 15.6 | 18.5 | 22.3 |
| RMSD | | | | |
| Bonds (Å) | 0.008 | 0.004 | 0.006 | 0.003 |
| Angles (°) | 0.93 | 0.60 | 0.69 | 0.64 |

**Table S3.** Crystallographic data collection and refinement statistics for **$\alpha^{Me}$A24**, **$\beta^{3}$K28**, **$\alpha^{Me}$K28**.

| | **$\alpha^{Me}$A24** | **$\beta^{3}$K28** | **$\alpha^{Me}$K28** |
|---|---|---|---|
| **PDB** | 9Z1T | 9Z1U | 9Z1V |
| **Data Collection** | | | |
| Unit cell dimensions (Å, °) | $a$ = 78.1, $b$ = 30.4, $c$ = 28.1, $\alpha = \gamma$ = 90, $\beta$ = 97.0 | $a$ = 84.9, $b$ = 30.9, $c$ = 28.1, $\alpha = \gamma$ = 90, $\beta$ = 102.3 | $a$ = 78.5, $b$ = 30.5, $c$ = 27.7, $\alpha$, $\gamma$ = 90, $\beta$ = 96.6 |
| Space group | C2 | C2 | C2 |
| Resolution (Å) | 50.00–1.60 (1.70–1.60) | 50.00–1.50 (1.60–1.50) | 50.00–1.65 (1.75–1.65) |
| Total observations | 139,759 | 52,562 | 63,968 |
| Unique observations | 8,775 | 11,517 | 7,979 |
| Redundancy | 15.9 (11.3) | 4.6 (2.8) | 8.0 (4.9) |
| Completeness (%) | 99.8 (100.0) | 99.5 (98.3) | 99.7 (98.9) |
| $I/\sigma$ | 54.8 (10.0) | 31.5 (6.5) | 29.4 (5.0) |
| $R_{meas}$ | 0.043 (0.285) | 0.031 (0.187) | 0.053 (0.312) |
| **Refinement** | | | |
| Resolution (Å) | 27.84-1.60 | 24.43–1.50 | 27.62-1.65 |
| $R$ | 0.175 | 0.175 | 0.196 |
| $R_{free}$ | 0.216 | 0.206 | 0.211 |
| Avg. B factor (Å$^2$) | 17.7 | 16.6 | 15.2 |
| RMSD | | | |
| Bonds (Å) | 0.011 | 0.009 | 0.006 |
| Angles (°) | 1.05 | 0.95 | 0.83 |
